# Local and Global Spatial Organization of Interaural Level Difference and Frequency Preferences in Auditory Cortex

**DOI:** 10.1093/cercor/bhx295

**Published:** 2017-11-09

**Authors:** Mariangela Panniello, Andrew J King, Johannes C Dahmen, Kerry M M Walker

**Affiliations:** Department of Physiology, Anatomy and Genetics, University of Oxford, Oxford, UK

**Keywords:** auditory cortex, binaural, ILD, mouse, 2-photon imaging

## Abstract

Despite decades of microelectrode recordings, fundamental questions remain about how auditory cortex represents sound-source location. Here, we used in vivo 2-photon calcium imaging to measure the sensitivity of layer II/III neurons in mouse primary auditory cortex (A1) to interaural level differences (ILDs), the principal spatial cue in this species. Although most ILD-sensitive neurons preferred ILDs favoring the contralateral ear, neurons with either midline or ipsilateral preferences were also present. An opponent-channel decoder accurately classified ILDs using the difference in responses between populations of neurons that preferred contralateral-ear-greater and ipsilateral-ear-greater stimuli. We also examined the spatial organization of binaural tuning properties across the imaged neurons with unprecedented resolution. Neurons driven exclusively by contralateral ear stimuli or by binaural stimulation occasionally formed local clusters, but their binaural categories and ILD preferences were not spatially organized on a more global scale. In contrast, the sound frequency preferences of most neurons within local cortical regions fell within a restricted frequency range, and a tonotopic gradient was observed across the cortical surface of individual mice. These results indicate that the representation of ILDs in mouse A1 is comparable to that of most other mammalian species, and appears to lack systematic or consistent spatial order.

## Introduction

The difference in sound level between the 2 ears provides an important sound localization cue for most mammals, and is the principal basis by which animals with small heads and high-frequency hearing, such as rats and mice, localize sounds in the horizontal plane (Chen et al. 1995; Wesolek et al. 2010; Lauer et al. 2011). Extracellular recordings have been used to explore the sensitivity of neurons to interaural level differences (ILDs) in the primary auditory cortex (A1) of a variety of species. Nevertheless, fundamental questions remain unanswered.

One important issue concerns the diversity of spatial tuning in A1. Studies of spatial and ILD sensitivity in a range of species have shown that the majority of A1 neurons prefer contralateral stimuli, though many are omnidirectional (i.e., lack spatial selectivity) and others respond best to ipsilateral or midline locations (e.g., Middlebrooks and Pettigrew 1981; Rajan et al. 1990; Irvine et al. 1996; Mrsic-Flogel et al. 2005; Campbell et al. 2006; Razak 2011; Zhou and Wang 2012). In contrast, it has been reported that neurons in rat A1 exhibit exclusively contralateral hemifield spatial preferences (Higgins et al. 2010; Yao et al. 2013). This raises questions about the nature of spatial tuning in A1 of other rodents, like mice, with comparable hearing ranges and head sizes.

A second, long-standing question concerns whether binaural preferences are spatially organized in A1. Early investigations of binaural sensitivity often categorized neurons discretely based on their responses to sounds presented monaurally or binaurally. Intriguingly, neurons in A1 of cats (Imig and Adrian 1977; Middlebrooks et al. 1980) and ferrets (Kelly and Judge 1994) were found to be organized into bands of binaural summation and suppression that alternate perpendicular to the tonotopic gradient and extend throughout the cortical depth, thereby providing evidence for columnar organization.

As later studies employed more diverse binaural response categories and a greater range of species, the spatial organization of binaural interaction properties within A1 became less clear. Rather than distinct bands, some studies reported spatial clusters of neurons with similar binaural properties scattered across the cortical surface (Reale and Kettner 1986; Kelly and Sally 1988; Recanzone et al. 1999; Rutkowski et al. 2000; Nakamoto et al. 2004), and others found organization only in thalamo-recipent layers (Phillips and Irvine 1983; Reser et al. 2000). The pallid bat appears to stand apart from other species by possessing 2 separate regions of A1: 1 in which neurons show ipsilateral ear inhibition, and another wherein neuronal responses are facilitated by binaural stimulation (Razak 2011). How neurons in mouse A1 are organized according to their binaural properties remains unexplored.

The aforementioned studies examined spatial response properties using microelectrodes, which sparsely sample the cortex, favor the most active neurons, and potentially pool responses from several neurons. If A1 neurons are arranged into binaural bands or clusters, neighboring neurons within those regions should have similar ILD preferences, but microelectrode recordings do not necessarily provide the spatial resolution to test this. In vivo 2-photon calcium imaging, on the other hand, can be used to simultaneously measure the responses of the majority of neurons located within a few hundred micrometers. The recent application of 2-photon calcium imaging to the mouse auditory cortex (Bandyopadhyay et al. 2010; Rothschild et al. 2010; Chen et al. 2011; Winkowski and Kanold 2013; Issa et al. 2014) has offered new insights into its tonotopic organization (Kanold et al. 2014). Furthermore, it has revealed fine gradients of binocular response preferences within the microarchitecture of ocular dominance columns in primary visual cortex (Kara and Boyd 2009). However, this high-resolution approach has not so far been used to probe the sensitivity of cortical neurons to binaural sound features.

Transgenic mouse strains allow cell-type specific targeting that is not yet possible in other mammals, and many of the new and most powerful technologies for studying neural circuits have been developed in this species (e.g., multiphoton imaging). Consequently, the mouse model is growing in popularity in studies of the auditory system, and it is important to establish how the representation of binaural cues in the mouse auditory cortex compares to other mammalian species.

In the present study, we used a genetically encoded calcium indicator and in vivo 2-photon imaging to characterize the cortical representation of ILD preferences at the microcircuit level in mice. By measuring the frequency tuning of these neurons, we were also able to compare the cortical organization of ILD and frequency preferences at both local and global scales.

## Materials and Methods

All animal procedures were approved by the local ethical review committee at the University of Oxford and performed under license from the UK Home Office. Twenty-one female mice were used in total: 18 C57BL/6 J mice (Harlan Laboratories); and 3 GCamP6f reporter line mice obtained by mating the floxed Ai95 (RCL-GCaMP6f)-D line (Jackson Laboratories; stock number 024105) with the CaMKIIalpha-Cre T29–1 line (Jackson Laboratories; stock number: 005359).

### Viral Vector Injection

GCamP6m expression was induced through viral vector injection in 15 C57BL/6J mice. At age 5–6 weeks, mice were premedicated with dexamethasone (Dexadreson, 4 μg s.c.), atropine sulfate (Atrocare, 1 μg s.c.) and carprofen (Rimadyl, 0.15 μg s.c.), and were then put under general anesthesia with fentanyl (Sublimaze, 0.05 mg/kg i.p.), midazolam (Hypnovel, 5 mg/kg i.p.) and medetomidine hydrochloride (Domitor, 0.5 mg/kg i.p.). Once anesthetized, the mouse was placed in a stereotaxic frame (Model 900LS, David Kopf Instruments). Body temperature was kept at 36–37 °C through the use of a DC Temperature Controller (FHC) with a rectal thermometer input and a heating mat output. The depth of anesthesia was monitored by rear paw pinch and through observation of the respiratory pattern. The eyes were covered with eye ointment (Maxitrol, Alcon) to prevent corneal desiccation. The skin over the injection site was shaved, cleaned with ethanol and injected with bupivacaine (Marcain, 40 μg s.c.). A scalp incision was then made and the right temporal muscle retracted to reveal the right upper squamosal bone of the skull. The location of A1 was estimated using stereotaxic coordinates (70% of the distance from Bregma to Lambda, and ~4.5 mm lateral from the midline) and 2 small holes (~0.4 mm diameter) were drilled over the right primary auditory fields, separated rostrocaudally by ∼0.5 mm. Vascular landmarks were also used to more precisely determine the location of the injection sites (Stiebler et al. 1997).

We used an adeno-associated viral vector expressing GCaMP6m under the synapsin I promoter (AAV1.Syn.GCaMP6m.WPRE.SV40, Penn Vector Core) to image calcium dynamics. Synapsins are neuron-specific proteins expressed in both excitatory and inhibitory cells (Kugler et al. 2003). Because ∼80% of cortical neurons are excitatory (White 1989), we expect the vast majority of the labeled cells to be excitatory neurons. Approximately 200 nl of the viral construct diluted (1:2) in phosphate-buffered saline (PBS, Sigma Aldrich) was slowly (~2 min) injected via each hole at 4 depths spanning 50–400 μm below the pial surface. Injections were made using a pulled glass pipette (20–30 μm inner tip diameter) and a custom-made air pressure injection system. Following injection, the skin was sutured close and general anesthesia was reversed with flumazenil (Anexate, 0.5 mg/kg i.p.) and atipamezol (Antisedan, 2.5 mg/kg i.p.). Postoperative buprenorphine (Vetergesic, 1 ml/kg s.c.) and enrofloxacine (Baytril, 2 ml/kg s.c.) were administered immediately after surgery and again 24 h later.

### Retrobead Injections

To confirm injection of the viral vector into the auditory cortex, we injected red fluorescent retrobeads (Lumafluor Inc, 2 μl undiluted) into 3 mice of the same age (5–6 weeks), following the same surgical procedure and the same injection coordinates described above. Retrograde labeling of thalamocortical neurons in the medial geniculate body, which is the main thalamic relay sending feed-forward inputs to auditory cortex, was later assessed using a fluorescent microscope.

### In Vivo 2-Photon Calcium Imaging

For the 15 GCaMP6m-injected animals, in vivo 2-photon imaging sessions were performed 3–6 weeks after the injection. All mice (15 GCaMP6m-injected and 3 from the reporter line) were imaged at <12 weeks of age, before the reported development of substantial high-frequency hearing loss (Ison et al. 2007). Mice were premedicated as above with dexamethasone and atropine. General anesthesia was induced with an intraperitoneal injection of 100 mg/kg ketamine (Vetalar) and 0.14 mg/kg medetomidine hydrochloride, and was maintained with hourly subcutaneous injections of ketamine (50 mg/kg/h) and medetomidine (0.07 mg/kg/h). After shaving the scalp and applying bupivacaine (40 μg s.c.), the skin was incised and the temporal muscle retracted to expose the viral vector injection sites. A circular craniotomy ∼2.5 mm in diameter was made over the right auditory cortex following the same stereotaxic coordinates and vasculature landmarks for all animals (see above). The exposed area was covered with a cover glass (Thermo Fisher Scientific), which was secured to the skull edge with cyanoacrylate adhesive (UltraGel, Pattex). Dental cement (UniFast Trad, GC Dental Products Corporation) was used to secure a small steel holding bar onto the left side of the skull and to create a small fluid basin around the craniotomy, in order to immerse the objective in artificial cerebro-spinal fluid during imaging. The mice were then head-fixed to the stage and placed under the microscope.

Imaging of calcium transients was performed using a Thorlabs B-Scope 2-photon microscope controlled by ScanImage 4.1 software (http://scanimage.org [last accessed 23 October 2017]). Excitation light was emitted by a Mai-Tai eHP laser (SpectraPhysics; 70 fs pulse width, 80 MHz repetition rate) tuned to 930 nm. The beam was directed into a Conoptics modulator (laser power, as measured under the objective, was 15–30 mW) and scanned through an 8 kHz resonant scanner in the *x*-plane and a galvanometric scanning mirror in the y-plane. The resonant scanner was used in bidirectional mode, at a resolution of 512 × 512 pixels, allowing us to acquire frames at a rate of ~30 Hz. A 16X/0.80W LWD immersion objective (Nikon) was used and emitted photons were guided through a 525/50 filter onto GaAsP photomultipliers (Hamamatsu). All neuronal fields were 250 × 250 μm in size and were imaged 200–350 μm below the pial surface, in cortical layers II/III (Anderson et al. 2009).

### Sound Presentation

All stimuli were presented to the ears via a closed acoustic delivery system comprising 2 EC1 electrostatic speakers (Tucker-Davis Technologies), each coupled to a 12-cm-long silicone tube leading into the ear canal. Delivery of the sounds directly into the ear canals provided precise control over ILDs and prevented acoustic cross-talk between the 2 ears. Sound stimuli were generated using a RZ6 multiprocessor (Tucker-Davis Technologies) and controlled via customized MATLAB (MathWorks) software. The output response of the speakers was measured using a Brüel & Kjær calibration system with a GRAS 40DP microphone coupled to the end of the silicone tube. The speaker output was then filtered to produce a flat spectral response (±3 dB) from 1.5–80 kHz at the desired sound levels.

All stimuli in this study were 100 ms in duration (~3 imaging frames) with 5 ms cosine onset and offset ramps, and were presented at a rate of 0.65 Hz. Broadband noise bursts (2–60 kHz) were presented binaurally with ILDs ranging from −30 dB to +30 dB, in 10 dB steps, where negative ILDs indicate that the sound level was greater in the contralateral (left) ear. This range covers the naturally occurring ILD range in mice (Chen et al. 1995; Lauer et al. 2011) and has been previously used to study ILD sensitivity in this species (Xiong et al. 2013). Based on the head-related transfer function (HRTF) of mice, the ILDs presented here correspond approximately to sounds coming from ∼0°, ±18°, ±61°, and ±90° in the azimuthal plane (Chen et al. 1995). However, a strict conversion of the ILD values of broadband sounds to the spatial directions of free-field stimuli is not possible because the generation of ILDs is frequency dependent. The same broadband noise bursts were also presented monaurally to each ear. We presented these stimuli at an average binaural level (ABL) of 60 and 80 dB sound pressure level (SPL) for 6 mice, and 40, 60, and 80 dB SPL for 12 mice (including the 3 mice from the reporter line). Diotic pure tones (100 ms duration, 5 ms onset/offset ramps) were also presented at 18 frequencies (1.9–50 kHz, with 0.6 octave spacing) and 4 levels (40, 60, 80, and 100 dB SPL). Ten repetitions of the tones and 12 repetitions of the noise stimuli were presented in pseudorandom order, in separate blocks. This stimulus set was repeated for each imaging field in each mouse.

The microscope and mouse were enclosed within a sound- and light-attenuating box. Ambient room noise was <42 dB SPL inside this box, and primarily consisted of energy <200 Hz, which is outside the hearing range of the mouse (Heffner and Heffner 2007). The resonant scanner generated a constant acoustical tone of 8 kHz during imaging that was <30 dB SPL near the mouse’s head. Cross-talk measurements between the speaker inputs to the 2 ears were made by inserting the calibration microphone in one ear canal and presenting a 95 dB SPL, 500-ms-long broadband noise burst to the contralateral ear. The intensity of the noise burst measured at the ear contralateral to stimulation was barely (<1 dB) above ambient room noise level.

### Histology

At the end of each imaging session, or 3 days after the fluorescent retrobeads injection, the mouse was overdosed (100 mg/kg ketamine and 0.14 mg/kg medetomidine, i.p.) and perfused transcardially, first with PBS and then with 4% paraformaldehyde in PBS. After 2 days in fixative, and one in 20% sucrose solution, the brain was sectioned in the coronal plane at a thickness of 60 μm on a freezing stage microtome and mounted on glass slides. The sections were examined with a Leica DMR upright fluorescence microscope. Excitation wavelength was 480 nm for visualizing eGFP and 530 nm for rhodamine retrobeads. Images were processed offline using ImageJ (NIH).

### Data Analysis

#### Isolating the Responses of Single Neurons from Imaging Fields

We analyzed data from 80 imaging fields (250 μm × 250 μm) across 18 mice. Analysis was performed offline using customized MATLAB software.

Mechanical drift in the *x*–*y* imaging plane was corrected using efficient subpixel registration methods (Guizar-Sicairos et al. 2008), and a time-averaged image was then generated from all recorded frames in the given field. Neurons were identified by visually inspecting this time-averaged frame and videos of frame-by-frame changes in fluorescence. Regions of interest (ROIs) corresponding to cell bodies were identified manually as in Barnstedt et al. (2015). The most common indicator of the health of a neuron labeled with a GCaMP indicator is the absence of nuclear filling (Tian et al. 2009; Chen et al. 2013). By manually identifying the imaged cell bodies, we were able to exclude all the filled cells from further analysis (as in Tian et al. 2009). For each cell, the signal at each time frame was calculated as the average fluorescence across all pixels inside the ROI (*F*(t)). A high pass filter with a cut-off of 0.02 Hz was applied to this time series to eliminate slow fluctuations in the signal. The baseline fluorescence (*F*_0_) for a given ROI was calculated as the median of the 10th–70th percentile of fluorescence values across all frames. The highest 30% of fluorescence values was not included in the baseline calculation in order to exclude the transient increases in calcium concentration due to action potentials. The fluorescence time series for the neuron was then corrected for the baseline using the formula: (*F*(t) – *F*_0_)/*F*_0_, which is commonly denoted as Δ*F*/*F*_0_. This normalizes the differences in overall fluorescence across cells that can occur due to differences in GCaMP6 expression. A correction for neuropil signal contamination was applied to each neuron’s fluorescence trace as described previously (Chen et al. 2013), using a contamination ratio of *r* = 0.6 (Kerlin et al. 2010).

#### Inferring Spikes from Calcium Transients

Spike trains were inferred from neuronal calcium transients using a compressive sensing technique extensively described in Dyer et al. (2013) and available on github (https://github.com/KordingLab/nerds [last accessed 23 October 2017]). Parameters for the estimation of calcium transient templates were set separately for GCaMP6m and GCaMP6f, as the rise and decay times differ for these 2 indicators. These parameters were optimized using our own dataset, and they agree well with those reported by Chen et al. (2013) for mouse V1 responses. The inferred spikes were used only to validate our findings presented here, which are all based on the analysis of the original calcium transients.

#### Identifying Responsive Neurons

We determined whether each neuron was significantly responsive to noise bursts and/or tones in separate analyses. The response to each presentation of a sound was compared across 2 time windows: the prestimulation period comprised of the 14 frames immediately prior to stimulus onset (~500 ms), and the stimulation period, which comprised the 14 frames from the onset of the stimulus (~500 ms). Paired *t*-tests (1-tailed, alpha = 0.01) were carried out on the maximum Δ*F*/*F*_0_ values in each of the pre and post-stimulus windows across repeated presentations of any one sound, for each ILD and each pure tone frequency. Neurons that responded significantly to the noise burst at any ILD were considered to be driven by noise bursts, and those responding to at least one of the 18 frequencies were considered to be responsive to tones. Expressing neural responses as the sum of Δ*F*/*F*_0_ values in each of these time windows, rather than the maximum Δ*F*/*F*_0_, yielded similar results to those reported here (data not shown).

Throughout all the analyses that follow, we defined the “response” of a neuron to a sound as the neuron’s difference in peak florescence between the stimulation and prestimulation period. By estimating the response as a difference between the values obtained in these time periods, we controlled for any temporally local fluctuations in fluorescence signals.

#### Quantifying ILD and Frequency Sensitivity in Responsive Neurons

We can think of each presentation of a sound as a single “trial” in our experiment. To assess the ILD sensitivity of neurons that were significantly responsive to noise bursts, a neuron’s trial-averaged response to each ILD and ABL combination was visualized as an ILD Response Area plot. To statistically test if a neuron was sensitive to ILD, the responses to binaural noise bursts were compared across the 7 ILDs using a 1-way ANOVA (alpha = 0.05). Sensitivity to ILD was assessed separately at each ABL.

If a neuron was found to be sensitive to ILD at a given ABL, its peak ILD was determined as the ILD eliciting the highest average response. Taking the peak of the response curve as the neuron’s stimulus preference has also been used in previous studies of ILD (Polley et al. 2013) and ITD (Belliveau et al. 2014) sensitivity in rodent auditory cortex. We calculated the slopes of the ILD response functions as the difference between the average neuronal responses to 2 contiguous ILD values. These slopes were calculated between all pairs of contiguous ILD values, and were derived separately for each ABL.

In addition to the peak ILD, we also calculated the weighted ILD preference of ILD-sensitive neurons. Weighted averaging has been used to define ILD or spatial preferences of neurons in previous studies (Mrsic-Flogel et al. 2005; Campbell et al. 2008; Middlebrooks et al. 1998), and has the advantage of quantifying a neuron’s ILD response based on the responses across the entire range of ILDs tested. We calculated the weighted ILD preference (also known as the “centroid”) of these neurons at each ABL as:

$${\mathrm{ILD}}_{\mathrm{weighted}}=\overset{n}{\underset{i=1}{\sum }}{\mathrm{ILD}}_{i}\;\cdot \;{\overset{®}{r}}_{i}/\overset{n}{\underset{i=1}{\sum }}{\mathrm{ILD}}_{i},$$

where *i* is the index of *n* = 7 ILD values, *ILD*_*i*_ is the signed *i*^th^ ILD value in dB, and ${\overset{®}{r}}_{i}$ is the trial-averaged neural response to *ILD*_*i*_.

Neuronal frequency sensitivity was also assessed for tone-responsive neurons. Frequency Response Area (FRA) plots were constructed from the average response to each tone frequency and ABL. The FRA was smoothed with a 3-point running average across frequencies to aid visualization. A 2-way ANOVA (with frequency and level as variables) was used to determine if the neuronal response was significantly modulated by sound frequency or level (alpha = 0.05). For neurons showing a main effect of frequency, the best frequency (BF) was then determined as the frequency eliciting the highest mean response, averaged across all levels (Guo et al. 2012; Barnstedt et al. 2015). If the neuron’s response at BF was <3 standard deviations above the prestimulus response, the neuron was classified as having no clear BF. Visual inspection of the FRA plots yielded similar BFs across the neural population (as in Rothschild et al. 2010; data not shown).

To identify the orientation of the BF gradient in each mouse, we followed the procedure described in Barnstedt et al. (2015). We correlated the BF of all neurons with their position on the *x* (rostro-caudal) axis. This correlation was recalculated while rotating the *x*-axis in 1° intervals throughout 360°. The axis rotation producing the strongest negative correlation was taken as the orientation of the tonotopic gradient.

#### Categorizing the Binaural Properties of Neurons

To facilitate comparisons with earlier papers describing the organization of binaural interactions in A1 (e.g., Imig and Adrian 1977; Middlebrooks et al. 1980), we discretely categorized the binaural properties of neurons based on their responses to noise bursts presented monaurally to either ear and diotically (i.e., with an ILD of 0 dB). As described above for ILD stimuli and pure tones, paired *t*-tests (1-tailed, alpha = 0.01) comparing the maximum Δ*F*/*F*_0_ values during the prestimulation and stimulation periods were used to determine if a neuron was responsive to each of the monaural and diotic noise stimuli. The results of these *t*-tests were then used to classify each neuron into 1 of 4 monaural categories, following the categorization used by Zhang et al. (2004). Neurons were categorized separately for each ABL. A neuron was classified as: (1) “EO” if it was responsive to monaural noise in the contralateral ear but not the ipsilateral ear; (2) “OE” if it was driven by monaural noise in the ipsilateral ear but not the contralateral ear; (3) “EE” if it was driven by monaural stimulation in both ears; (4) “OO/F” if it responded only to the diotic stimulus, but not monaural stimulation, thus showing binaural facilitation (termed “PB” in Zhang et al. 2004).

The OO/F category describes a form of binaural interaction. For the other 3 categories, we determined whether each neuron showed binaural interactions by computing a Binaural Interaction Index (BII). This index divides the response elicited by the diotic stimulus by the sum of responses to the 2 monaural stimuli (Goldberg and Brown 1969; Zhang et al. 2004; Higgins et al. 2010). Following the conventions of Zhang et al. (2004), a diotic response that was 20% higher than the sum of the 2 monaural responses (i.e., a BII > 1.2), was considered to indicate “binaural facilitation”. Such neurons were categorized as EO/F, OE/F or EE/F, depending on their monaural response category. A BII < 0.8 was considered to represent “binaural inhibition”, and these neurons were categorized as EO/I or OE/I. If 0.8 < BII < 1.2, the binaural interaction was very weak, possibly resulting from random fluctuations. In these cases, neurons were categorized as EO/N, OE/N or EE/N, where “N” indicates no binaural interaction. Finally, EE neurons with a diotic response <80% of the sum of the monaural responses and >80% of the response to monaural stimulation of the dominant ear were considered to exhibit “binaural occlusion”, and were classified as EE/O.

#### Principal Components Analysis

We used principal components analysis to identify common patterns in responses to noise bursts that differ in ILD across a large population of neurons. The population included all neurons that were responsive to noise bursts at any ABL (paired *t*-test, alpha = 0.01), pooled across the 9 mice for which broadband stimuli were presented at 3 ABLs. The principal components analysis was carried out on a response matrix consisting of the trial-averaged responses to the 21 stimuli (7 ILDs × 3 ABLs). The average responses of each neuron were normalized by the neuron’s maximum average response across all ILDs, so that neurons with higher overall spike rates (i.e., larger fluorescence transients) did not dominate the population variance.

#### Decoding ILD Responses

Our decoding algorithm was adopted from the opponent-process decoder described in detail by previous extracellular recording studies (Stecker et al. 2005; Keating et al. 2015). For each noise-responsive neuron (paired *t*-test, see above), responses to noise at a given ILD were pooled across all ABLs presented, so that the decoder was required to classify ILD and not just the absolute level in either ear. We performed linear regression to determine whether each neuron’s response showed an overall increase or decrease with more positive ILD values. Negative regression slopes indicated that the neuron preferred contralateral ILDs, while those with positive slopes preferred ipsilateral ILDs.

In order to normalize differences in peak fluorescence (i.e., spike rates) across neurons, the trial-averaged responses of all monotonic neurons to each ILD were divided by the maximum average response across all ILDs. For a given population size (*s*), neurons were then sampled at random from all GCaMP6m-injected mice and imaging fields. The normalized neural responses to each ILD were averaged across all contralateral-preferring neurons and all ipsilateral–preferring neurons, separately. This produced an ILD response curve for the contralateral- and ipsilateral preferring neural populations (see example in Fig. 5*B*). The difference between these 2 population responses provided a population-wide ILD opponent-channel code. While nonsimultaneous neuronal responses were pooled here across different imaging fields, previous studies have shown that noise correlations have minimal effects on the performance of this type of opponent-channel decoder (Keating et al. 2015).

Our decoding algorithm attempted to identify the ILD of the sound presented to the mouse on each individual “trial” by comparing the opponent-channel response on that trial to the average opponent-channel responses to ILDs derived from all other trials (i.e., leave-one-out cross-validation). The ILD producing the most similar opponent-channel response was determined to be the ILD of the trial in question. The performance of this decoder was quantified for each mouse using the mean unsigned error, calculated as average absolute difference between the true ILD and the decoded ILD across trials. The mean unsigned error was normalized by the maximal error possible for the task.

The opponent-channel decoder was run 1000 times at each tested population size, and the resulting mean unsigned error was averaged across these repetitions to remove the effects of the specific subpopulation of neurons randomly sampled for decoding.

#### Estimating Noise and Signal Correlations Between Pairs of Neurons

Signal correlation was estimated to determine the degree to which a pair of neurons showed similar tuning to sound properties. A related metric, noise correlation, was also calculated for each neuronal pair to estimate the correlation between the activity of the 2 neurons that is unrelated to the type of sound presented, but which may instead arise due to network dynamics and connectivity between the neurons. Signal and noise correlations were calculated separately for responses to ILD noise stimuli and tones. In each case, only neurons showing a significantly driven response and sensitivity to the stimulus parameter of interest (see *t*-test and ANOVA descriptions above) were included in the correlation analyses.

We followed the procedures for calculating noise and signal correlations previously described in detail by Rothschild et al. (2010), and so they are summarized only briefly here. To calculate noise correlations, each response of a neuron to a single sound presentation was normalized for stimulus effects by subtracting the neuron’s average response to that stimulus type across multiple presentations (i.e., the given ILD or frequency, at the given ABL). The noise correlation between a pair of neurons was then estimated as the correlation between the normalized trial-by-trial responses of the 2 neurons. Signal correlations were estimated as the correlation between the trial-averaged responses of any 2 neurons, minus the noise correlation estimate for the neuronal pair. Signal and noise correlation could, therefore, only be calculated between neurons that were simultaneously measured in the same imaging field.

A bootstrapped data shuffling approach was used to test the statistical significance of our observed noise and signal correlations, and to compare how the distributions of these values would look for neural responses that are statistically independent. During noise correlation bootstrapping, the trial-to-trial responses were randomly drawn (with replacement) from the neuron’s responses to all repetitions of the given stimulus. During signal correlation bootstrapping, responses on each trial were randomly drawn (with replacement) from responses to all stimuli. The average noise and signal correlations across 1000 iterations of this shuffling procedure produced a distribution of noise and signal correlations, which we used to test the statistical significance of the signal and noise correlations in our unshuffled dataset (paired *t*-test, alpha = 0.01). Repeating the bootstrapping for 500 and 2000 iterations produced similar results (data not shown).

#### Cluster Analysis

We used a bootstrapping approach to test if neurons that were located proximally in 2D cortical space had more similar preferences for sound frequency and ILD compared to neurons located further apart. In this manuscript, we define “clustering” as this statistical tendency for nearby neurons to show more similar stimulus preferences than distant neurons.

To test for ILD response clustering, we calculated the “ILD preference distance” as the absolute difference between the weighted ILD preferences of a pair of neurons. For a given mouse, the ILD preference distances were calculated for all pairs of neurons that were separated by ≤150 μm. We then calculated the ILD preference distances of the same number of randomly chosen pairs of neurons that were located >150 μm apart in that mouse (as in Rothschild et al. 2010 and Schnupp et al. 2015). The mean ILD preference distances from each of 1000 of such randomly chosen samples of neuronal pairs served as our bootstrapped estimate of the ILD preference similarity of distant neurons. We adopted an alpha of 0.05. Therefore, if the average ILD preference distance for the local pairs of neurons was below the fifth percentile of the bootstrapped values for distant pairs, this indicated a significant spatial clustering of ILD preferences in this mouse.

This bootstrapped analysis was used to test for clustering of weighted ILD preferences, BFs, and binaural categories. For the latter, we specifically tested clustering of EO/I, EO/F, and OO/F cells, as these were by far the most common categories observed. These categories were coded as −1, 0, and 1 for the clustering algorithm. We repeated these analyses at spatial bin sizes of 100, 150, 200, and 250 μm, and found that the greatest amount of clustering for all 3 stimulus parameters occurred at 150 μm. Therefore, only the results of 150 μm binning are reported in the Results. The mouse A1 covers an area of approximately 1 mm × 0.25 mm across the cortical surface, with the tonotopic gradient oriented along its longest axis (Kanold et al. 2014).

Clustering of weighted ILD preferences and binaural categories was only tested for neurons with a BF ≥ 10 kHz. This choice was based on evidence showing that, in anechoic conditions and using free-field stimuli, ILDs produced by low-frequency sounds (<10 kHz) in the mouse are negligible (Chen et al. 1995; Allen and Ison 2010; Lauer et al. 2011).

## Results

### Targeting Primary Auditory Cortex

**Figure 1.**
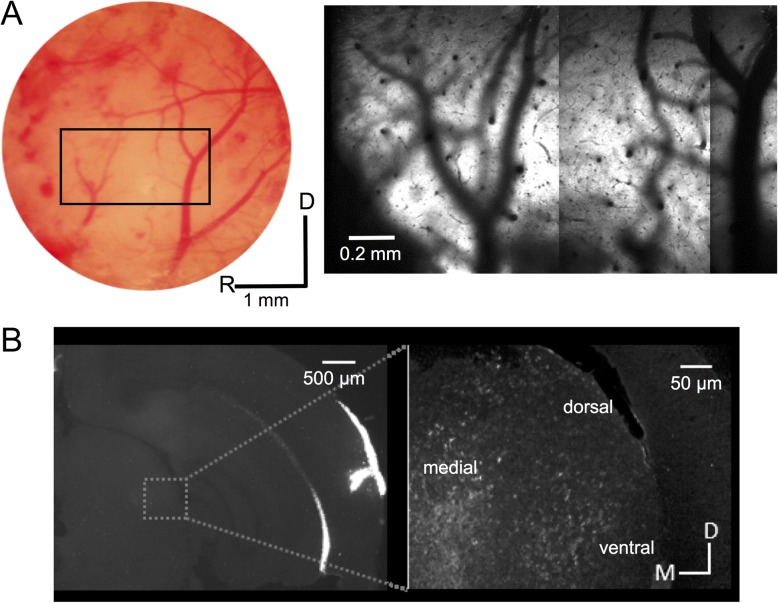
Anatomical confirmation of auditory cortex targeting. *(A)* A picture of the cortical surface of one mouse, showing the areas were A1 and AAF are located. The detail shows a widefield 2-photon image of the same area, labeled with GCaMP6m. *(B)* A coronal section from a mouse in which red fluorescent retrobeads were injected into the auditory cortex following the same coordinates used for GCaMP6m injections. The inset shows labeling in the medial geniculate body at higher magnification. The locations of different divisions (dorsal, medial, ventral) are indicated. D, dorsal; M, medial; R, rostral.

To investigate the micro-organization of spatial tuning in layers II/III of mouse auditory cortex, we performed in vivo 2-photon calcium imaging in 15 animals in which the calcium indicator GCaMP6m had been expressed by injected AAV. Auditory cortex was targeted for viral injection by following previously described stereotaxic coordinates (see “Materials and Methods” section). We also used vascular landmarks to target the area extending between 2 major V-shaped vessels running ventrodorsally between the temporal and the parietal lobes, which has been shown to correspond to the core auditory regions of A1 and AAF (Tsukano et al. 2016) (Fig. 1*A*).

To verify the location of these AAV injections, we injected retrograde fluorescent microbeads at the same coordinates in a further 3 mice. The resulting pattern of retrograde labeling in the medial geniculate body of the thalamus confirmed that our injections had successfully targeted auditory cortex. Labeling was found mainly in the medial and ventral divisions of the medial geniculate body (Fig. 1*B*), indicating that primary and non-primary fields had been included in the injection sites. Furthermore, the BFs of the imaged neurons in individual mice expressing GCaMP6f transgenically exhibited a consistent high-to-low, rostral-to-caudal gradient, which fits with the tonotopic organization of mouse A1 (Hackett et al. 2011; Guo et al. 2012; Issa et al. 2014; Joachimsthaler et al. 2014). In the neighboring anterior auditory field, the tonotopic gradient has a different orientation, while secondary fields either contain neurons with higher or unclassified BFs, or are not tonotopically organized (Stiebler et al. 1997; Hackett et al. 2011; Guo et al. 2012; Issa et al. 2014; Tsukano et al. 2015).

In 2 mice we injected GCaMP6m ∼1 mm more rostrally than our usual coordinates, and in 2 of our transgenic animals we imaged some fields beyond A1 outside these coordinates. In these regions, we observed that fewer neurons (13%) demonstrated significant frequency tuning (ANOVA, *P* < 0.05) than in A1 (37%), and that FRAs showed less clear V-shaped tuning curves. We calculated the “coefficient of BF variation” as the standard deviation of BF across neurons in the same imaging field (Issa et al. 2014), in order to quantify the cotuning of neighboring neurons. This coefficient was slightly higher in regions outside A1 (1.023 ± 0.18 octaves, mean ± standard deviation for injected mice; 1.205 ± 0.82 octaves for transgenics) than at our A1 coordinates (0.922 ± 0.42 octaves for injected mice; 0.778 ± 0.51 octaves for transgenics), but these values were not significantly different (2-sample *t*-test; *P* > 0.05). The BF variation was, as expected, slightly higher than that obtained in the central nucleus of the inferior colliculus of anesthetized mice using the same indicator (0.727 ± 0.43; Barnstedt et al. 2015). Thus, while we cannot rule out the possibility that our imaging fields occasionally extended beyond the primary auditory field, the frequency tuning of the neurons and their organization into a single tonotopic gradient suggest that the majority of the data presented here were from A1.

### Neuronal Sensitivity to ILD

**Figure 2.**
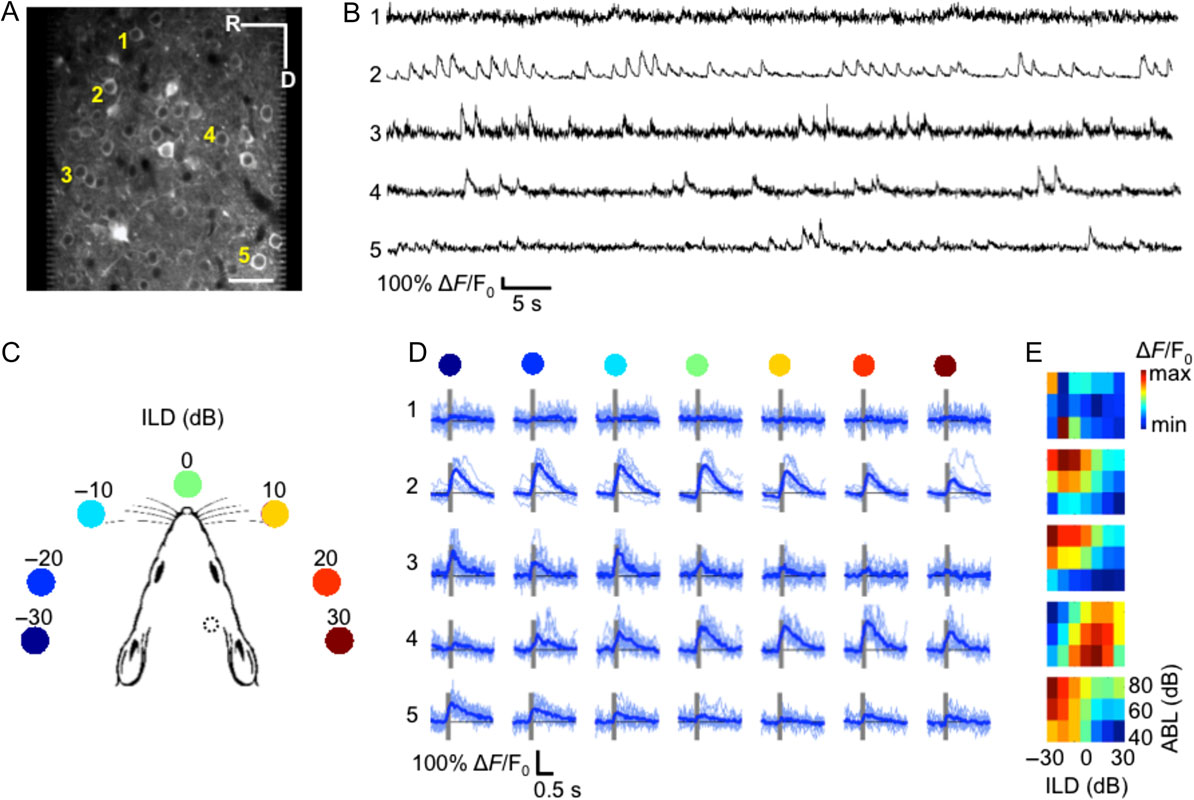
ILD sensitivity in mouse auditory cortex demonstrated using in vivo 2-photon calcium imaging of GCaMP6m. *(A)* An example imaging area at 290 μm below the pial surface. Scale bar is 50 μm. *(B)* The lines plot the relative changes in fluorescence of the 5 neurons indicated in *(A)* during presentation of noise bursts. (*C)* Cartoon representation of auditory stimuli: 7 ILDs ranging from −30 to 30 dB ILD in 10 dB steps were presented to the mouse during the imaging session via calibrated speakers coupled to the ears via tubes inserted into the ear canals. The position of the colored dots indicates the approximate corresponding azimuthal angle relative to the mouse head based on acoustical measurements by Chen et al. (1995). Monaural stimuli were also presented to each ear (not shown). The dotted circle indicates the position of our cranial window for imaging. *(D)* Each row shows the individual (light blue) and mean (dark blue) ∆*F*/F_0_ values of the same 5 neurons from *(A,B)* in response to each of the 7 ILD values, for noise bursts presented at 60 dB ABL. Gray bars indicate stimulus presentation time. The colored dots from *(C)* are shown above each column for reference. *(E)* ILD response area plots for each of the 5 neurons shown in *(A,B,D)*. The color scale indicates the trial-averaged response of the neuron to each of the 7 ILDs (x-axis) presented at each of 3 ABLs (rows). R, rostral; D, dorsal.

We imaged calcium transients from 1913 neurons in 61 imaging fields with an area of 250 × 250 μm (Fig. 2*A*,*B*), across 15 mice expressing GCaMP6m via injected AAV. While imaging, we presented 12 repetitions of broadband noise bursts with ILDs of 0, 10, 20, or 30 dB favoring either the ipsilateral or the contralateral ear (Fig. 2*C*), as well as monaural broadband noise bursts in each ear. Each of these 9 stimuli were presented at either 2 (60 and 80 dB SPL, *n* = 6 mice) or 3 (40, 60, and 80 dB SPL; *n* = 9 mice) ABLs.

The majority of calcium transients were well aligned with the onset of the stimulus (Fig. 2*D*; Supplementary Fig. 1A). Some neurons showed a clear preference for one of the ILDs presented, exhibiting consistent responses across repetitions of the same stimulus (e.g., neurons 4–5, Fig. 2*D*). For other neurons, the magnitude of the stimulus-evoked calcium transients varied considerably across repetitions of the same ILD (e.g., neuron 2, Fig. 2*D*), while some did not respond to these stimuli (e.g., neuron 1, Fig. 2*D*). For each neuron, we represented the average response to the noise bursts at each ILD and ABL using ILD response area plots (Fig. 2*E*; Supplementary Fig. 1*A,B*). Most responsive neurons showed V-shaped ILD response areas, indicating monotonic intensity sensitivity, tuning to a particular ILD, and sharper ILD preferences at lower sound levels (e.g., neurons 2, 3, and 5, Fig. 2*E*). A smaller subset of neurons showed different tuning characteristics, such as suppression by more intense sounds (e.g., neuron 4, Fig. 2*E*). As the examples in Fig. 2*D,E* illustrate, neurons in the same imaging field often had heterogeneous ILD preferences.

To rule out the possibility that the ILD responses we observed were biased by the presence of sub-threshold signals in the analysis, and to test the reliability of calcium signals in our dataset, we inferred firing rates from the intracellular calcium transients in a subset of our data. As expected, the ILD response curves obtained using the height of calcium signals and inferred spikes were similar (Supplementary Fig. 2). We therefore used the height of the calcium signals to quantify the stimulus selectivity of the neurons throughout the rest of our analyses.

**Figure 3.**
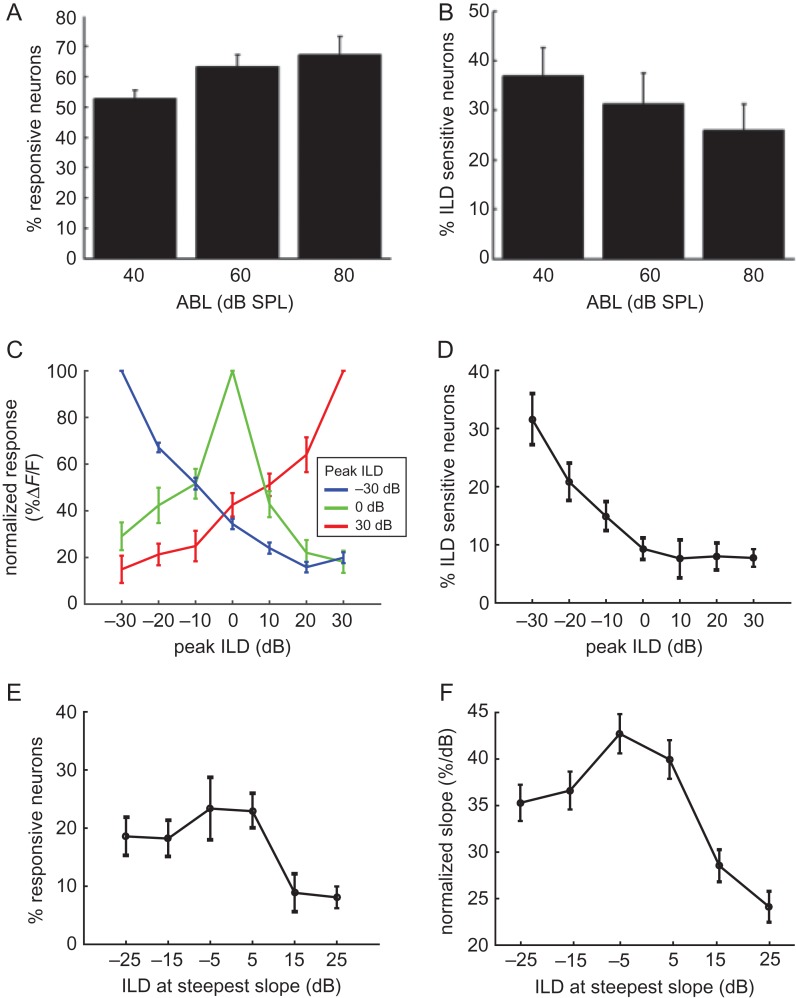
The majority of ILD-sensitive neurons prefer broadband stimuli favoring the contralateral ear. (*A)* Mean percentages of neurons responsive to sound stimulation (t-test; *P* < 0.01) across mice at each of the ABLs tested. (*B)* Mean percentages of neurons showing ILD sensitivity (ANOVA; *P* < 0.05) across mice. (*C)* Average normalized ILD response functions for all neurons responding maximally to ILDs of −30 (blue), 0 (green) or 30 dB (red) (data obtained from responses at 60 dB ABL). (*D)* Mean percentages of neurons preferring different ILD values at 60 dB ABL across all mice injected with GCaMP6m. (*E)* Mean position of the steepest ILD response function slopes at 60 dB ABL. (*F)* Variation in mean ILD response function slope, normalized across all the noise-responsive neurons, with ILD. All error-bars indicate standard errors.

We found that noise bursts presented at 40 dB ABL elicited significant responses (paired *t*-tests; *P* < 0.01) from about half of our imaged neurons (52.3 ± 3.6%; mean ± standard error across mice) (Fig. 3*A*), while most neurons responded to noise presented at 80 dB ABL (62.5 ± 4.8%). This indicates a larger recruitment of neurons for higher sound intensities over the 40–80 dB range. On the other hand, the proportion of cells that were significantly modulated by ILD (ANOVA; *P* < 0.05) fell slightly as the ABL was increased from 40 dB (18.7 ± 3.7% of all the imaged cells; 37.3 ± 5.6% of noise-responsive cells) to 80 dB SPL (16.1 ± 3.7% of all imaged cells; 25.7 ± 4.9% of noise-responsive cells) (Fig. 3*B*). Overall, 32% of responsive neurons were sensitive to ILD for at least one ABL, which is similar to the proportions reported from intracellular recordings in rat auditory cortex (Kyweriga et al. 2014).

The ILD preference of a neuron can be simply summarized as the ILD producing the largest response, which we term the “peak ILD”. However, this value does not describe the response of the neuron to other ILDs or whether, for example, the neuron has a monotonic response curve or is sharply tuned to one ILD value. To visualize the typical ILD response curve of neurons with a given peak ILD, we normalized the responses of each neuron to its maximum response, and then averaged these normalized responses across neurons with the same peak ILD. Figure 3*C* shows the average ILD response curves for neurons with a peak ILD of −30 dB (blue), 0 dB (green), or 30 dB (red). The ILD functions for neurons preferring −30 and 30 dB ILDs had steep monotonic slopes and were almost symmetrical to one another around the midline. Neurons preferring 0 ILD were sharply tuned, but this response curve was asymmetrical around the midline, favoring responses to contralateral stimuli.

Figure 3*D* shows the percentage of neurons peaking at each ILD, averaged across mice. The ILD functions of most neurons peaked at ILDs corresponding to contralateral positions at all ABLs tested (Fig. 3*D* and Supplementary Fig. 3*A*), and a clear minority preferred sounds that were more intense in the ipsilateral ear (22.6% of neurons at 60 dB ABL; Fig. 3*D*). In order to determine the region of ILD “space” within which neuronal responses were most informative, we calculated the slope of the response curve between contiguous ILD values. This slope is steepest at ILDs for which the neuron is most informative. At 60 dB ABL, the majority of neurons were most informative about ILDs around the midline or favouring the contralateral ear (Fig. 3*E*). The distribution was flatter at 80 dB ABL, perhaps due to the reduced ILD sensitivity at this high sound level (Supplementary Fig. 3*B*). We also calculated, at each ILD, the average slope across all neurons with their maximal slope at that ILD. This analysis showed that ILD response slopes peaking near 0 dB were more sharply tuned than slopes at more “peripheral” ILDs (Fig. 3*F*).

**Figure 4.**
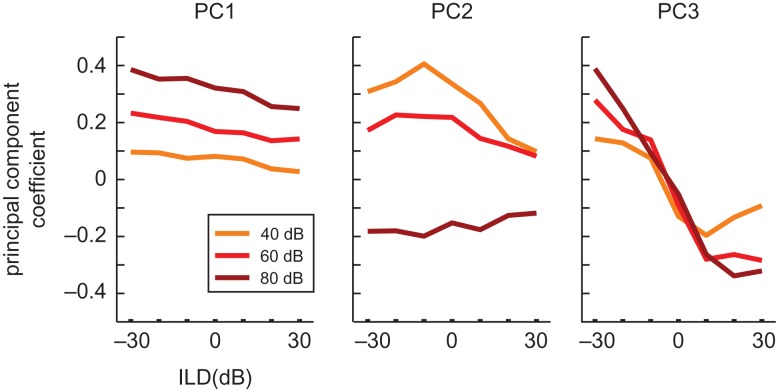
Principal components analysis of the neural population responses. The eigenvectors of the first (left), second (center), and third (right) principal components of all noise-responsive neurons are plotted as a function of stimulus ILD. Sounds presented at the 3 different ABLs are plotted separately and color-coded. This demonstrates the effects of ILD and ABL explained by each principal component.

The large number of neurons recorded in 2-photon imaging experiments makes principal components analysis a useful tool for capturing common response trends across neurons. We used principal components analysis to identify patterns in the responses of noise-responsive auditory cortical neurons across the 7 ILDs and 3 ABLs presented. This provides a description of ILD response curves that is free from any preconceived expectations. The analysis produces 21 principal components, ordered according to the amount of neural response variance that they explained. The first 3 principal components together were sufficient to account for 78.3% of the variance in the neural responses (53.7, 16.6, and 8% each), and the coefficients of these principal components are plotted in Figure 4. The first principal component illustrates that a monotonic change in the neural response across ABL, together with a more modest monotonic ILD response, explains the most variance in our data. The second component describes responses that are tuned to a particular ILD at the quietest sound level, but are omnidirectionally inhibited at the highest sound level tested. Finally, the third principal component describes monotonic ILD response functions, in which neurons respond preferentially to sounds in one hemifield in a level-independent manner. Together, these results suggest that noise-responsive neurons in mouse auditory cortex commonly: (1) are monotonically facilitated (or sometimes inhibited) by increases in overall sound level; and (2) have monotonic ILD response curves.

**Figure 5.**
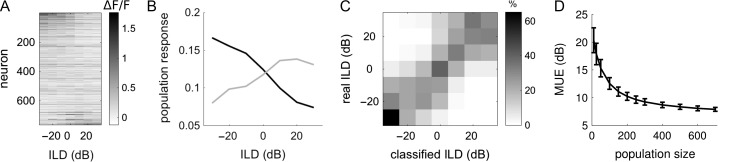
Decoding stimulus ILD from neural responses using an opponent-channel algorithm. (*A*) Trial-averaged responses (764 neurons, across 9 mice) to noise bursts that vary in ILD. (*B*) ILD response curves averaged across 2 neural subpopulations: those preferring contralateral ILDs (black), and those preferring ipsilateral ILDs (gray). (*C*) Joint distribution of the real ILD of the stimulus (rows) and the ILD classification of the decoder (columns). The grayscale represents the percentage of trials corresponding to each real/classified combination. (*D*) The mean unsigned error (MUE) of the decoder is shown across increasing neural population sizes.

The above analyses demonstrate that a subpopulation of mouse auditory cortical neurons encode ILDs and usually prefer ILDs that favor the contralateral ear. We next sought to investigate whether a decoder is able to classify the ILD of sounds presented on single trials based on the neural population response, irrespective of the absolute level of the sound. This type of decoding analysis tests whether ILD population codes are sufficiently distinguishable across ILDs and reliable across trials to support sound localization behavior. We employed an opponent-channel decoding algorithm (see “Materials and Methods” section), which has been successfully used to investigate population codes for auditory space in the auditory cortex of cats (Stecker et al. 2005) and ferrets (Keating et al. 2015). The opponent-channel decoder classifies ILDs by comparing the relative responses to a sound from 2 populations of neurons: one preferring sounds that are greater in the left ear, and the other preferring sounds that are greater in the right ear. A linear regression analysis showed that 88% of all ILD-sensitive neurons in our dataset (40% of all noise-responsive neurons) showed an overall decrease or increase in response strength across the range of ILDs tested (*P* < 0.05) (Fig. 5*A*). The majority of our ILD-sensitive neurons could therefore be described as having monotonic ILD response functions. The responses of individual neurons were normalized and pooled into either the ipsilateral or contralateral subpopulation (Fig. 5*B*). The ILD of a sound presented on a given trial was then classified based on the relative activation between 2 subpopulations of these neurons on that trial (Fig. 5*B*).

We found that the opponent-channel decoder was able to classify the sound ILD on single trials with good accuracy, despite the fact that the sounds were presented across a wide range of ABLs (Fig. 5*C*). Naturally, the decoder performed more poorly when the classification was based on a small population of neurons, but the normalized mean unsigned error plateaued at ~9 dB (± 0.5 dB standard deviation) with a population of 300 neurons (Fig. 5*D*).

### Local Spatial Distribution of Binaural Preferences

**Figure 6.**
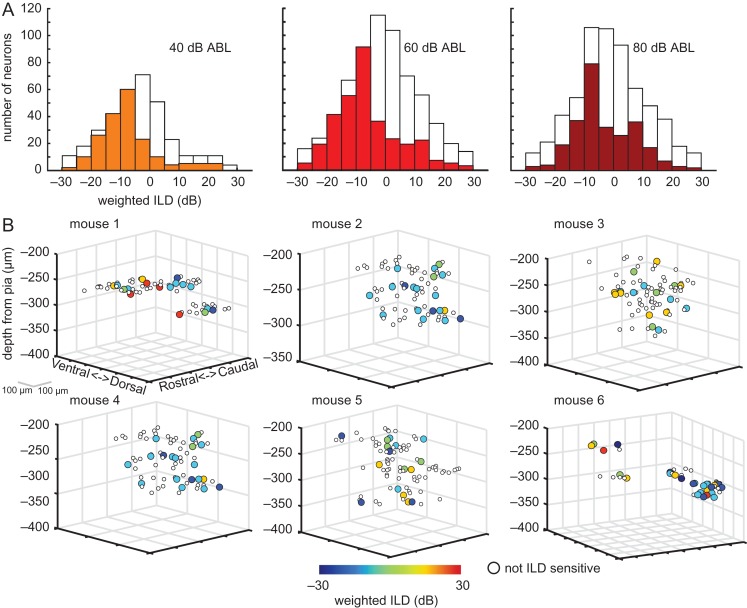
The local spatial distribution of weighted ILD preferences is heterogeneous. (*A)* Weighted ILD preference distributions obtained from all ILD-sensitive neurons across all mice injected with GCaMP6m. Data are shown separately at the 3 ABLs tested. The white bars in the plots indicate the same distributions, but obtained for neurons that were not responsive to noise bursts. (*B)* Neurons from 6 mice are plotted according to their position along the rostro-caudal, dorso-ventral, and cortical depth. ILD-sensitive neurons are color-coded according to their weighted ILD preference. For these plots, data points were obtained from responses at 60 dB ABL.

Previous studies have used a weighted average (or “centroid”) of responses when examining the distribution of spatial preferences of auditory neurons (Middlebrooks et al. 1998; Mrsic-Flogel et al. 2005; Campbell et al. 2008), and so we adopted this metric here. For a control comparison, we first plotted the weighted ILD preferences of neurons that were nonresponsive to noise bursts (*t*-test, *P* ≥ 0.01; white bars in Fig. 6*A*). As expected from the lack of a significant stimulus-evoked response, these values were normally distributed around the midline. In contrast, the weighted ILD preferences of most ILD-sensitive (ANOVA, *P* < 0.05) neurons were skewed heavily towards the contralateral ear, especially for quieter ABLs.

We examined the distribution of weighted ILD preferences within a local region of cortex. In most cases, AAV transfection of neurons extended over about 300 μm rostrocaudally and dorsoventrally. The location of each noise-responsive neuron was plotted as a circle on the rostro-caudal and dorso-ventral axes, with the color of the circle indicating the cell’s weighted ILD preference (Fig. 6*B*). Despite the overall prevalence of contralateral and midline preferences, these maps illustrate that even neighboring neurons in the same imaging field (250 μm × 250 μm) could have widely different ILD preferences (e.g., mice 1 and 6, Fig. 6*B*). In some mice, we observed more homogeneous ILD preferences. For example, the majority of neurons in mice 2 and 4 (Fig. 6*B*) preferred contralateral ILDs. However, no systematic spatial organization of weighted ILD preferences was apparent in these maps across the 15 mice we investigated. We used a bootstrapping approach to test empirically, at each ABL, whether neurons with similar ILD preferences were spatially clustered in each mouse (see “Materials and Methods” section). The values were not found to significantly cluster in any the 15 mice. Together, these results suggest that there is no obvious spatial organization of weighted ILD preferences in layer II/III of mouse auditory cortex within the local (250 μm × 250 μm) microcircuit.

**Figure 7.**
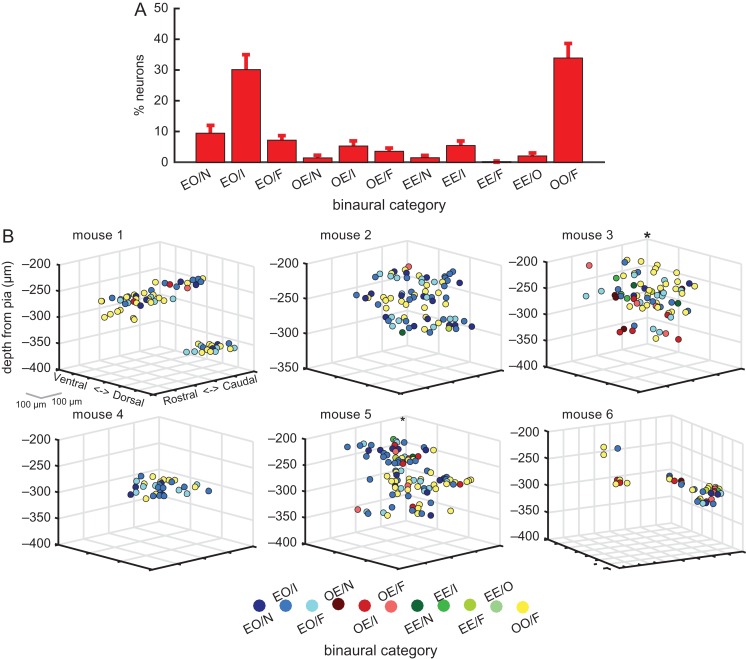
Local spatial distribution of binaural categories. (*A)* Percentages of responsive neurons in each binaural category, averaged across mice (mean ± sem; 60 dB ABL). (*B)* Relative cortical location of neurons in the same 6 mice shown in Figure 6*B*. Neurons are color-coded according to their binaural category (see “Materials and Methods” section for details). In these plots, binaural categories for each neuron were derived from the response at 60 dB ABL.

In addition to measuring ILD sensitivity, we categorized each neuron based on its responses to noise bursts presented monaurally in the ipsilateral ear, monaurally in the contralateral ear, and diotically at 0 dB ILD. Figure 7*A* shows the distribution of binaural classes across all noise-responsive neurons in 15 mice, tested at 60 dB ABL (similar results were obtained at 40 and 80 dB ABL, Supplementary Fig. 4). The vast majority of cells showed either binaural activation with no monaural response (OO/F neurons; 33.9 ± 4.7%), or a response to monaural contralateral stimuli but not to monaural stimulation of the ipsilateral ear (EO neurons; ∼47%). Of the neurons classified as EO, the majority showed binaural inhibition (EO/I; 30.1% ± 4.5% of all neurons). Similarly, the majority of OE neurons also showed binaural inhibition, although these were a small minority of the whole neuronal population (OE/I; 5.3% ± 1.4%). Overall, only 12% of neurons belonged to categories showing no binaural interactions (namely, EO/N, OE/N or EE/N).

Previous extracellular recording studies in cat A1 have described bands of neurons with similar binaural properties that run orthogonal to the isofrequency axis and which are separated by ~0.5–1 mm (Imig and Adrian 1977; Middlebrooks et al. 1980). Given that cat A1 is ~8 mm in length along the tonotopic axis, while the full extent of mouse A1 is only ~1 mm, we can expect to see evidence of such binaural bands within our 250 × 250 μm imaging window. This might manifest as either: (1) a predominance of one type of binaural category within an imaging field that differs across fields, (2) clear spatial segregation of 2 or more binaural categories within a field, or (3) gradients of ILD preferences, akin to the gradients of binocular disparity and ocular dominance observed at the microcircuit level in cat V1 (Kara and Boyd 2009). The local spatial distribution of these binaural categories within the cortex is plotted in Figure 7*B*, and can be compared to the weighted ILD preference distribution of the same neurons in Figure 6*B*. As seen with the ILD preferences, the binaural categories were often heterogeneously distributed within a local region of the cortex. However, in most imaging fields there was a disproportionally large number of EO/I, EO/F, or OO/F neurons. The bootstrapping cluster analysis for responses at 60 dB ABL showed that neurons belonging to one of these 3 binaural classes were likely to be located near (within 150 μm) neurons with the same class in only 3 out of 15 mice (among these, mice 3 and 5 in Fig. 7*B*). Therefore, we again found little evidence for local spatial clustering of binaural response properties in layer II/III of mouse auditory cortex.

### Comparisons of Binaural and Frequency Preferences within the Local Microcircuit

**Figure 8.**
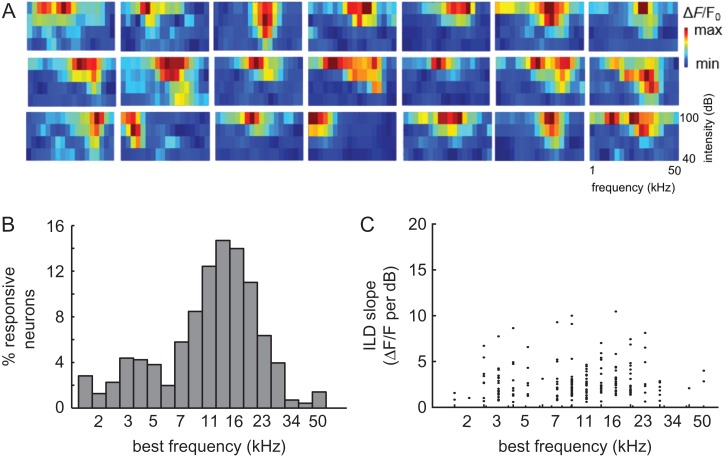
Local spatial distribution of BFs. (*A)* Frequency response areas from a sample of 21 neurons imaged in the 6 mice shown in Figures 6*B* and 7*B*. (*B)* BF distribution for all neurons in the dataset. (*C)* Steepest ILD response function slope of each neuron plotted as a function of its BF.

The distribution of pure tone frequency preferences has previously been characterized in mouse auditory cortex using 2-photon calcium imaging (Bandyopadhyay et al. 2010; Rothschild et al. 2010; Issa et al. 2014). Frequency tuning, therefore, provides a useful benchmark for our current investigation of the spatial organization of ILD preferences. In addition to the stimuli used to characterize the binaural sensitivity of the neurons, we presented pure tones at frequencies ranging from 1.9 to 50 kHz in 0.27 octave steps (spanning ~4.5 of the 6 octaves audible to mice; Stiebler et al. 1997) and at 4 intensity levels (40, 60, 80, and 100 dB SPL). Among all imaged neurons (*n* = 1765), 42.7% were responsive to tones (paired t-test, *P* < 0.01) and 65.6% of these responsive neurons were sensitive to tone frequency (2-way ANOVA, *P* < 0.05). Most of the frequency-sensitive neurons had V-shaped FRAs (Fig. 8*A*), with narrower tuning at lower sound levels. In keeping with electrophysiological recording studies of mouse auditory cortex (Linden et al. 2003; Joachimsthaler et al. 2014), we observed an over-representation of BFs from ~10 to 28 kHz (Fig. 8*B*). Previous 2-photon calcium imaging studies of mouse A1 (Rothschild et al. 2010; Issa et al. 2014) and of corticocollicular terminals (Barnstedt et al. 2015) have also reported a similar distribution of BFs. Evidence for whether our C57BL/6 mice would have experienced any high-frequency hearing loss by the age at which we imaged them is conflicting (e.g., Willott et al. 1993; Ison et al. 2007). The BFs in our dataset covered almost the full range of those obtained for thalamocortical axon terminals in a C57BL/6 strain in which the Cdh23^ahl^ allele that otherwise predisposes this strain to age-related high-frequency hearing loss has been corrected (Mianné et al. 2016; Vasquez-Lopez et al. 2017), but these strains may differ in the proportions of neurons tuned to high frequencies.

High-frequency neurons rely on ILDs for their spatial selectivity, whereas low-frequency neurons are generally more sensitive to interaural time differences (Imig and Adrian 1977; Campbell et al. 2006). However, a comparison of the maximum ILD slope and BF for all neurons that were sensitive to both ILD and frequency (*n* = 496 neurons at 60 dB ABL) revealed no correlation between these 2 measures (*r* = 0.023, *P* = 0.75) (Fig. 8*C*).

We examined the degree of similarity in stimulus sensitivity (“signal” correlations) and in stimulus-independent activity (“noise” correlations) between simultaneously imaged pairs of neurons, spaced up to 250 μm apart. Noise correlations can arise from connections within a local network of cells, causing them to fire together. The signal and noise correlations were estimated separately using broadband noise and pure tone stimulation, allowing us to compare the local spatial organization of ILD and frequency sensitivity with a common metric.

**Figure 9.**
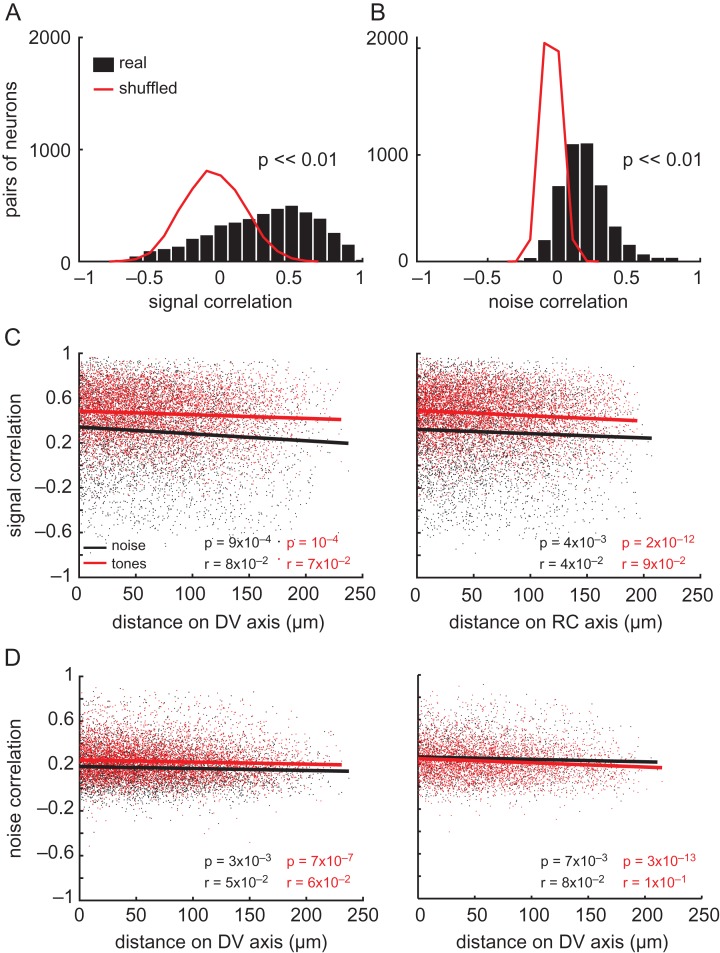
Comparisons of binaural and frequency preferences within the local cortical microcircuit. (*A)* Distributions of pairwise signal correlations during ILD stimuli presentation are shown for real (black bars) and shuffled (red line) data. The *p* value is indicated for a paired t-test between the 2 distributions. (*B)* Data presented as in (*A)*, but for noise correlations. (*C*) Signal correlations calculated during presentation of ILD stimuli (black dots and line) and pure tones (red dots and line) as a function of dorso-ventral (left) and rostro-caudal (right) cortical distance between simultaneously imaged neuronal pairs. (*D)* Noise correlations presented as in *C*. In (*C)* and (*D)*, lines indicate the best linear fit to the data.

Signal and noise correlations in noise responses were carried out only on those neurons whose responses were significantly modulated by ILD (*n* = 600 neurons). As the distribution in Figure 9*A* shows, the mean signal correlation in these responses, after removing the effects of trial-to-trial noise correlations, was highly variable (0.3 ± 0.35, mean ± standard deviation; *n* = 4278 neuron pairs). Noise correlations were less variable than signal correlations, and mainly ranged from 0 to 0.4, with a mean value of 0.18 ± 0.16 (Fig. 9*B*). Shuffling these data in a bootstrapping analysis (red lines in Fig. 9*A,B*), demonstrated that the signal and noise correlations we observed were significantly larger than chance (*P* << 0.01).

We also computed the signal and noise correlations during pure tone stimulation for pairs of frequency-sensitive, simultaneously imaged neurons (*n* = 6327 pairs). Signal (Fig. 9*C*) and noise (Fig. 9*D*) correlations were then plotted for both tones (red) and broadband noise (black) stimuli as a function of the distance between neuronal pairs along the dorso-ventral (left) and rostro-caudal axes (right). These plots allowed us to investigate whether cells positioned more proximally in cortical space showed more similar responses than distant neurons. The signal correlations (0.46 ± 0.22) for tone responses were much higher than those measured in the responses to ILD stimuli (*t*-test, *P* << 0.01), possibly reflecting the more homogeneous distribution of BFs relative to the preferred ILDs within local regions of the cortex. The noise correlations for pure tone responses (0.24 ± 0.15, mean ± standard deviation) were similar to those exhibited by pairs of neurons during presentation of the ILD noise stimuli, and to those reported in previous studies of auditory cortex (Rothschild et al. 2010). A tail of higher noise correlation values (up to 0.8) was present among proximal neurons, both during presentation of ILD stimuli and pure tones, suggesting that nearby neurons were more likely to be active on the same trials. Noise and signal correlations decreased only modestly, although significantly, with distance along the rostro-caudal and dorso-ventral axes, as indicated by the *r* and *P* values in Figure 9*C* and *D*. The fact that these correlations remain high and stable throughout the imaging field is consistent with the presence of local (~200 μm) processing networks in auditory cortex, as proposed by Rothschild et al. (2010).

**Figure 10.**
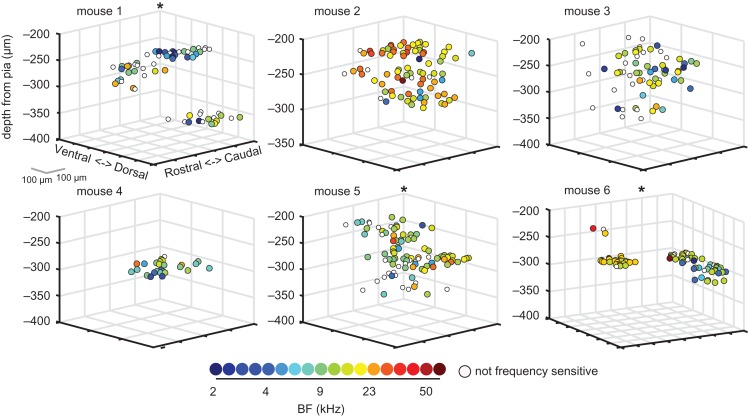
Neuronal sensitivity to pure tone frequency is more homogeneously distributed than sensitivity to ILDs. Three-dimensional plots of the relative cortical location of neurons in the same mice shown in Figures 6*B* and 7*B*. Neurons are color-coded according to their BF. Tone-responsive neurons that did not show significant frequency sensitivity are shown in white.

Visual inspection of 3D plots of neuronal BFs throughout cortical space confirmed that within a local region of A1, neurons were often tuned to a common frequency range (Fig. 10). Some imaged areas contained predominantly low-frequency neurons (mouse 1, Fig. 10), whereas others were dominated by higher frequency tuning (mouse 2, Fig. 10). Our bootstrapped cluster analysis indicated that the BFs of neurons were spatially clustered within these local fields in 7 out of 15 mice (including mice 1, 5, and 6 in Fig. 10). The absence of significant clustering in the other mice may be due to the limited cortical area in which neuronal BFs were measured in those mice. This is the case, for example, in mouse 4 (Fig. 10).

### Global Spatial Distribution of Binaural and Frequency Preferences

We have so far focused on the distribution of binaural and frequency sensitivity within local areas (≤250 × 250 μm) of auditory cortex. The availability of a reporter mouse line expressing GCaMP6f in cortical neurons constitutionally expressing the excitatory marker CaMKII alpha enabled us to examine the representation of such preferences over a much larger area (up to ~1.2 × 1.2 mm) in 3 mice. This also allowed us to target A1 by observing the high-to-low, rostral-to-caudal tonotopic gradient that distinguishes this field from surrounding areas of auditory cortex in mice (Hackett et al. 2011; Guo et al. 2012; Issa et al. 2014).

Out of a total of 992 imaged cells in these transgenic mice, 40.9% were found to be responsive to noise bursts (across the 3 ABLs) and 32% of these noise-responsive neurons were sensitive to ILDs. For pure tone stimulation, 26.4% (262 neurons) of the imaged neurons were found to be responsive and 51.9% (136 neurons) of these neurons were sensitive to tone frequency. We found a slightly larger proportion of neurons to be responsive to noise and tones in our experiments that used GCaMP6m (see above) compared to GCaMP6f, which is to be expected given the increased sensitivity of the higher-affinity “m” form of GCaMP6 (Chen et al. 2013). Similarly, the mean signal (0.21 ± 0.15; mean ± standard deviation) and noise (0.11 ± 0.07) correlations between pairs of simultaneously imaged neurons during pure tone presentation were lower when measured with GCaMP6f. Importantly, despite the doubling of the pairwise correlations overall in GCaMP6m animals, the relative difference between the signal and noise correlations and between the correlations measured for the 2 stimulus types remained the same across these 2 indicators.

To the best of our knowledge, this is the first time a reporter line expressing GCaMP6f has been used to study auditory cortical responses in mice: for this reason, we cannot directly compare our percentages of responsive and sensitive cells to any previous report. However, Chen et al. (2013) described the performance of GCaMP6f when studying orientation selectivity in the visual cortex of the mouse and found that ~35% of imaged cells were responsive to visual stimulation, which is very similar to the proportion found here for auditory stimulation in A1.

**Figure 11.**
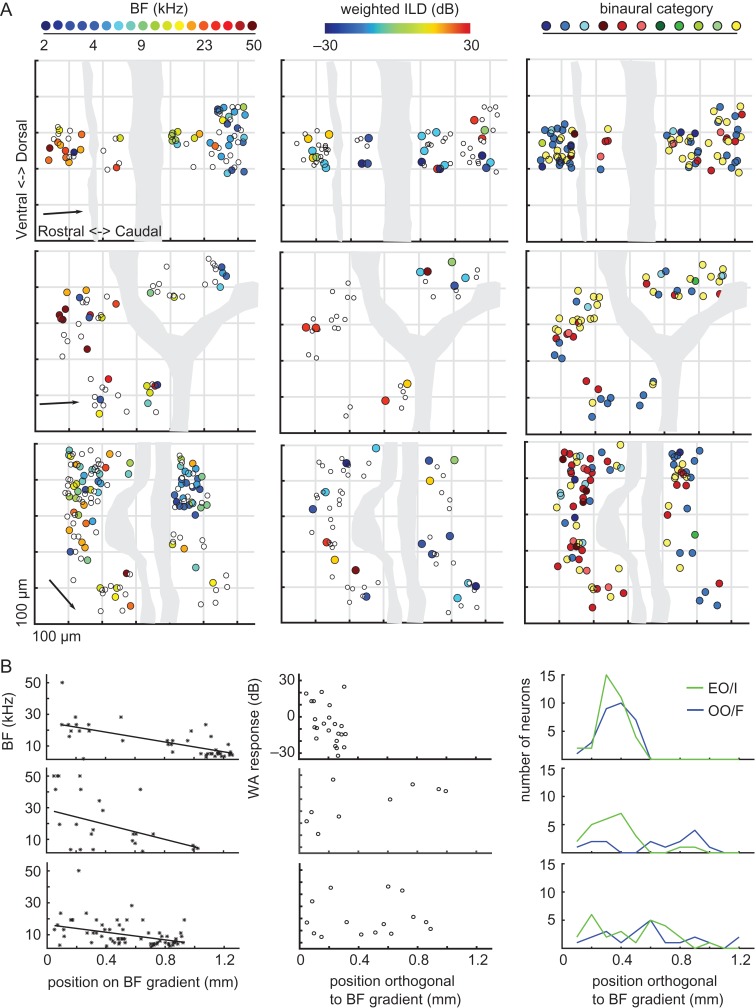
Sound frequency, but not binaural preferences, are topographically organized over large distances in A1. (*A)* Each plot shows the location of neurons along the rostro-caudal and dorso-ventral axes of the cortex. Data from a different transgenic mouse expressing GCaMP6f are plotted in each row, with neurons color-coded according to their BF (left column), weighted ILD preference (middle column), or binaural category (right column). Dotted grids indicate 200 × 200 μm. Gray shaded areas illustrate the position of blood vessels on the cortical surface. In the BF plots, the arrow indicates the orientation of the tonotopic gradient. (*B)* BFs plotted against distance along the tonotopic axis in each mouse, together with the corresponding line of best fit. (*C)* Weighted ILD preferences (WA, weighted average) plotted against distance along the axis orthogonal to the tonotopic gradient. (*D)* Number of EO/I and OO/F cells at different positions along the axis orthogonal to the tonotopic gradient.

For each mouse, we produced 2D maps in which the BFs of the neurons were represented according to their positions along the rostro-caudal and dorso-ventral axes of the cortex (Fig. 11*A*, left column). As expected for A1, a gradient of low to high-frequency preferences was observed in all 3 mice, proceeding from the most caudal to the most rostral imaging fields. For each mouse, the orientation of the tonotopic gradient was identified analytically (see “Materials and Methods” section), and is indicated by the arrows in Figure 11*A* (left column). Plotting the BF of each neuron as a function of distance along this axis confirmed the existence of a smooth tonotopic gradient in all animals (Fig. 11*B*). Because, the same brain coordinates were imaged in all animals in this study, the presence of this tonotopic gradient confirmed the targeting of A1 in both the 3 transgenic mice and the 15 GCaMP6-injected mice. The large cortical area imaged in our transgenic mice favored the identification of local clusters of BFs in our bootstrapped analysis, and statistically significant BF clustering was confirmed in all 3 transgenic animals (*P* < 0.05).

In contrast to the topographic order of sound frequency representation, maps of weighted ILD preferences in the transgenic mice suggested that this parameter did not change systematically across the cortical surface (Fig. 11*A*, middle column). The medial border of ILD response functions (based on analyses in Razak 2011) also showed no clear spatial organization within the cortical microarchitecture (<250 μm) or at the global scale (~1 mm) (Supplementary Fig. 5). We calculated the coefficient of variation for weighted ILD preferences similarly to the coefficient of BF variation presented above (see *Targeting primary auditory cortex*). Very similar values were obtained with GCaMP6m in injected mice (16.17 ± 1.2 dB, mean ± standard deviation) and GCaMP6f in transgenic mice (15.11 ± 3.3 dB, mean ± standard deviation) (2-sample *t*-test; *P* > 0.05).

A prevalence of EO/I, EO/F, and OO/F neurons was evident in these transgenic mice (Fig. 11*A*, right column), as in the injected mice. Furthermore, visual inspection of the binaural category maps in these animals revealed some patches of binaural clusters, amid an overall heterogeneous distribution. Some previous electrophysiological studies have suggested that neurons sharing similar binaural properties form bands or clusters along the isofrequency axis at this global scale, so we examined these trends in our data. First, we plotted the distribution of preferred stimulus locations along the axis that was orthogonal to the BF gradient in each mouse (Fig. 11*C*). This plot revealed no systematic relationship between the weighted ILD preferences of these neurons and their cortical position. Second, the bootstrapped cluster analysis also failed to find statistical evidence of weighted ILD preference clustering in these 3 mice. Finally, we plotted the number of OO/F and EO/I neurons within 100 μm bins along the isofrequency axis (Fig. 11*D*). These neuron types often overlapped along this spatial axis, and a possible spatial segregation of categories was apparent in only 1 of the 3 mice examined (middle plot). However, the bootstrapped analysis provided no evidence for statistically significant clustering of EO/I, EO/F, and OO/F cells in these 3 mice.

Together, these results suggest that on the global scale, a clear tonotopy is evident in mouse A1, whereas no clear segregation of neurons with different binaural properties is present beyond occasional clustering of binaural properties.

## Discussion

We used in vivo 2-photon calcium imaging to investigate ILD sensitivity and the local and global organization of binaural responses in layer II/III neurons of mouse auditory cortex. Approximately one-third of acoustically-responsive neurons showed significant ILD sensitivity and most of these responded monotonically to values corresponding to contralateral free-field sound sources, in keeping with microelectrode recording results in other mammalian species (Middlebrooks and Pettigrew 1981; Campbell et al. 2006). Nevertheless, ILD sensitivity varied considerably across individual neurons, and included peaked ILD functions corresponding to midline locations and neurons with a preference for ipsilateral ear stimulation. Amid this diversity, our cortical maps and statistical clustering algorithm found little evidence for anatomical clustering of neurons with similar ILD sensitivity (0/18 mice showed significant clustering) or of the same binaural category (3/18 mice were significantly clustered) at the 250 μm scale, and no evidence for a broader (>0.5 mm) systematic organization of these properties across the cortex. This contrasts with tone frequency preferences, which showed significant clustering in over half of mice imaged (10/18), and a clear tonotopic gradient (3/3 transgenic mice). Together with the spectral cues generated by the head and external ears (Lauer et al. 2011), ILDs determine the spatial selectivity of the neurons and therefore indicate that, as in other species (Middlebrooks et al. 2002), there is no map of space in mouse auditory cortex.

### ILD Sensitivity

Our analysis of ILD sensitivity is consistent with the presence of a majority of neurons responding more vigorously at higher ABLs (Fig. 2*E*, 3*A*, 4, and Supplementary Fig. 1), and preferring contralateral ILDs (~65% of noise-responsive neurons; Fig. 2*E*, 3*D*, 4, 5, 6, 11*A,C*, and Supplementary Fig. 1). Neurons preferring stimuli centered at the midline (0 dB ILD; ~10% of noise-responsive neurons) or in the ipsilateral ear (~24% of noise-responsive neurons) were also present (Fig. 2*E*, 3*D*, 5, 6, 11*A, C*, and Supplementary Fig. 1). A similar range of ILD sensitivity has been reported in electrophysiological studies of auditory cortex in adult mice (Polley et al. 2013), rats (Higgins et al. 2010; Kyweriga et al. 2014), cats (Irvine et al. 1996; Zhang et al. 2004), ferrets (Campbell et al. 2006), pallid bats (Razak 2011), and monkeys (Zhou and Wang 2012). This may underpin the heterogeneous spatial receptive fields of high-frequency A1 neurons reported in many species (Middlebrooks and Pettigrew 1981; Imig et al. 1990; Rajan et al. 1990; Mrsic-Flogel et al. 2005; Razak 2011). It is also interesting to note that we observed an enhanced response to ILDs favoring the ipsilateral ear at the highest sound level used (80 dB SPL; Supplementary Fig. 1). Consistent with this result, fMRI studies of human auditory cortex, which tend to present sounds at ~80 dB SPL to avoid masking by scanner noise, also report ILD functions with a secondary maxima at 30 dB ipsilateral (Stecker et al. 2015; Higgins et al. 2017). Together, these results indicate that range of ILD response functions observed in the auditory cortex of mice is typical of those found in other mammalian species with good high-frequency hearing, so this is a highly conserved and presumably important coding strategy for spatial sound representation.

We found that about one-third of responsive neurons were ILD-sensitive (Fig. 3*B*). This fits with the results of intracellular recordings in rat auditory cortex (Kyweriga et al. 2014), whereas extracellular recordings of ILD sensitivity in other mammals have found a higher percentage of spatially sensitive neural units (Irvine et al. 1996; Rutkowski et al. 2000; Nakamoto et al. 2004; Campbell et al. 2006). This could reflect a true species difference, as carnivores have generally been found to have better sound localization acuity than most rodents on behavioral tasks (Heffner et al. 1994). However, extracellular recording techniques tend to oversample particularly active neurons (Shoham et al. 2006) and are more prone to pooling action potential responses across multiple neurons, which could help to explain the discrepancies across these groups of studies.

A recent neural decoding study has suggested that the presence of heterogeneous spatial tuning across individual neurons within a cortical hemifield may be essential to sound localization performance (Goodman et al. 2013). We used a previously established opponent-channel decoder to examine how well the responses of mouse auditory cortical neurons can support ILD classification (Fig. 5). We found that with large populations of neurons in our dataset (>200 neurons), the decoder performed with a mean unsigned error of ~9.2 dB (±0.5 dB standard deviation). This is approximately double the mean unsigned error of ~4.5 dB reported for 200 ferret auditory cortical neurons using the same algorithm (calculated from the normalized errors in Keating et al. 2015), which fits with the poorer minimal audible angles reported in mice (Heffner et al. 2001; Lauer et al. 2011; but see Allen and Ison 2010). In the free field, monaural spectral cues will also contribute to broadband sound localization in mice, but it is interesting to note that cortical sensitivity to ILD cues alone is a good predictor of the azimuthal sound localization performance measured in ferrets with narrowband noise stimuli (Keating et al. 2015). Our results therefore provide testable predictions for future sound localization experiments in mice.

As in ferrets (Campbell et al. 2006) and cats (Stecker et al. 2005), we found that the slopes of the ILD response functions were usually sharpest around the midline (Fig. 3*E*, Supplementary Data Fig. 3*B*) at the 2 lower ABLs tested. This supports the evidence from several species that sound localization accuracy is greatest near the midline (Mills 1958; Parsons et al. 1999; Recanzone and Beckerman 2004). Behavioral studies of sound localization in mice are so far limited (Ehret and Dreyer 1984; Heffner et al. 2001, Lauer et al. 2011), and it is unknown whether their acuity varies with stimulus eccentricity in the manner predicted by the ILD sensitivity of cortical neurons.

Studies quantifying the HRTF of mice have found that ILDs are negligible at low frequencies (Chen et al. 1995; Lauer et al. 2011), which suggests that only neurons with high BFs would prefer large ILDs. However, we found no relationship between BF and sensitivity to ILDs (Fig. 8*C*), in keeping with previous results in rat A1 (Yao et al. 2013). There are several explanations for this result. First, ILDs show weaker frequency dependence in echoic environments (Mlynarski and Jost 2014), and are larger overall for sound sources that are very close to the head (Duda and Martens 1998). Consequently, nearby low-frequency sounds in more natural, echoic environments may generate larger ILDs in mice than those described by traditional free-field studies in this species. In support of this prediction, it has been shown that low-BF neurons in the brainstem of the cat exhibit sensitivity for ILDs (Tollin and Yin 2005). Second, A1 neurons are known to be highly non-linear, and respond to energy in broadband sounds that is well outside the excitatory frequency response area estimated with pure tones (Linden et al. 2003). In fact, 56.9% of ILD-sensitive neurons in our study were found to be insensitive to the frequency of pure tones. Thus, even low-BF neurons can respond preferentially to large ILDs.

### Organization of Binaural Preferences

The 1D tonotopic organization of A1 and some other cortical fields potentially allows a systematic variation in sensitivity to other stimulus features within the isofrequency domain (Schreiner and Winer 2007). Among the sound parameters that may be arranged in this fashion, bands of cells with similar binaural properties elongated approximately orthogonal to the isofrequency axis have been described in A1 (Imig and Adrian 1977; Middlebrooks et al. 1980) and the secondary auditory cortical area (Schreiner and Cynader 1984) in cats. Although later work employing more complex schemes for classifying binaural interactions failed to identify these binaural bands, evidence that cortical neurons with the same binaural properties form anatomically discrete clusters has been obtained in cats (Reale and Kettner 1986; Nakamoto et al. 2004), ferrets (Phillips et al. 1988; Kelly and Judge 1994), rats (Kelly and Sally 1988), guinea pigs (Rutkowski et al. 2000), owl monkeys (Recanzone et al. 1999), and pallid bats (Razak 2011).

In the present study, we adopted a binaural categorization accounting for both neuronal responses to monaural stimulation and for binaural interactions. This choice was made in order to facilitate comparisons with the above literature. We found that the majority of responsive cortical neurons could be classified as either EO/I, OO/F, or EO/F (Fig. 7*A*). The proportions of these cells in our data are highly similar to those reported in an intracellular study of rat auditory cortex (Kyweriga et al. 2014). The demonstration of cortical binaural facilitation (OO/F) in the mouse is an interesting result in itself, as it suggests that the mouse may be used to study binaural mechanisms for attending to signals in noisy environments. In fact, we found that 88% of neurons in our injected mice showed some form of binaural interaction. This is consistent with previous results in rodents (Higgins et al. 2010; Polley et al. 2013), as well as in higher mammals (Middlebrooks et al. 1980; Zhang et al. 2004; Kitzes 2008), which also show that most responsive neurons exhibit binaural interactions.

We found that the binaural classes of neurons were spatially distributed throughout the cortex in a largely unorganized manner (Fig. 7 and 11*A,D*). Statistically significant local clustering of EO/I, EO/F, and OO/F cells was observed in only 3 of 18 animals. In these 3 cases, the clustering did not resemble the 2 large, anatomically separate regions described in pallid bats (Razak 2011) and rat (where they reside in A1 and VAF, respectively; Higgins et al. 2010). While it remains possible that clearer evidence for anatomically segregated neurons with distinct ILD response functions may exist outside the regions mapped in the present study, the scattered clusters of binaural cell types we observed do resemble those described in earlier studies in rats (Kelly and Sally 1988), cats (Zhang et al. 2004), ferrets (Kelly and Judge 1994), and monkeys (Recanzone et al. 1999). A possible origin for the segregation of binaural response properties lies in the parallel brainstem pathways to cortex, which predominantly utilize either excitatory or inhibitory binaural interactions to derive sensitivity to ITDs and ILDs, respectively. However, whereas other species use both binaural cues for localization in the horizontal plane, mice are primarily dependent on ILDs (Chen et al. 1995; Wesolek et al. 2010; Lauer et al. 2011). This may therefore explain the lack of binaural bands in cortex, although this explanation is inconsistent with the segregation of binaural responses in the rat auditory cortex (Kelly and Sally 1988).

As in previous studies (e.g., Middlebrooks et al. 1998; Higgins et al. 2010; Yao et al. 2013), we used both the ILD eliciting the largest response (i.e., the peak ILD) and the weighted average of ILD responses (or weighted ILD preference), as measures of spatial preference. But whereas a systematic shift in activity across A1 with stimulus ILD or azimuth has been described in pallid bats (Razak 2011), the present experiments show that preferred ILDs are heterogeneous within the local microarchitecture of mouse auditory cortex and are not spatially organized on a more global scale. It remains possible that binaural properties are more clearly organized within or between higher cortical areas, or within deeper layers of mouse A1.

### Methodological Considerations

Two-photon calcium imaging enabled us to measure the responses of large numbers of neurons within the supragranular layers of auditory cortex, while knowing their cortical location with unprecedented spatial resolution. However, due to the slow buffering of calcium from neurons following spiking activity, it is not possible to resolve the fine timing of individual action potentials using this technique. Several electrophysiological studies have demonstrated that the timing of action potentials generated by auditory cortical neurons can sharpen the spatial tuning provided by a firing rate code (Stecker and Middlebrooks 2003; Nelken et al. 2005), but the functional relevance of this timing information remains unclear. Both spike rate and first-spike latencies of neural responses are informative about a sound-source’s spatial location (Mickey and Middlebrooks 2003; Mrsic-Flogel et al. 2005; Nelken et al. 2005), but sound localization accuracy in ferrets can be well accounted for by an opponent-channel decoder that uses only the firing rates of A1 neurons (Keating et al. 2015).

Bandyopadhyay et al. (2010) and Rothschild et al. (2010) used 2-photon calcium imaging of bulk loaded fluorescent dyes in anesthetized mice to show that the BFs of nearby neurons in layers II/III of A1 can differ substantially, with tonotopy becoming apparent only at larger spatial scales. Subsequent 2-photon imaging studies revealed that frequency selectivity is locally more homogeneous in layer IV of A1 (Winkowski and Kanold 2013), whereas Issa et al. (2014) reported robust tonotopy even in layers II/III of A1 in awake transgenic GCaMP3 mice. Our experiments were carried out under anesthesia so that precisely calibrated binaural stimuli could be delivered to the ears, to maximize head stability during imaging, and to facilitate comparison with previous studies of ILD processing. Although we cannot rule out the possibility that anesthesia may have contributed to the heterogeneity of ILD sensitivity we observed (Fig. 6*B*, Fig. 11*A,C*), the spatial tuning of auditory cortical neurons in awake cats has been found to be more level invariant but otherwise largely unchanged relative to anesthetised cats (Mickey and Middlebrooks 2003). Moreover, although we observed local variability in BF, the majority of neurons within local cortical regions were tuned to a comparable frequency range (Fig. 10) and tonotopic gradients were observed in individual mice (Fig. 11*A,B*). It therefore seems unlikely that the use of anesthesia alone can account for the much less precise organization of binaural response properties in the cortex. Rather, it appears that the ILD preferences of A1 neurons are more varied within imaging fields than their BFs, as indicated by the marked differences in signal correlations.

There are, however, several caveats to this conclusion. First, our calcium imaging was restricted to cortical layers II/III. Some authors have reported that binaural properties vary with depth and that a clustered organization may be present only in the thalamorecipient layers (Phillips and Irvine 1983; Reser et al. 2000). Indeed, we observed that the predominant binaural category in an imaging field can change with depth, even within the supragranular layers. Second, 2-photon imaging studies have shown that the highly ordered pinwheel organization of orientation tuning in the primary visual cortex of higher mammals is absent in rodents (Ohki and Reid 2007). Consequently, high-resolution calcium imaging in non-rodent species will be needed to confirm whether the functional organization of binaural and other auditory response properties is preserved across mammals.

## Supplementary Material

Supplementary Data

## References

[bhx295C1] Anderson LA, Christianson GB, Linden JF 2009 Mouse auditory cortex differs from visual and somatosensory cortices in the laminar distribution of cytochrome oxidase and acetylcholinesterase. Brain Res. 1252:130–142. 19061871 10.1016/j.brainres.2008.11.037

[bhx295C2] Allen PD, Ison JR 2010 Sensitivity of the mouse to changes in azimuthal sound location: angular separation, spectral composition, and sound level. Behav Neurosci. 124:265–277. 20364886 10.1037/a0018913 PMC2856490

[bhx295C3] Bandyopadhyay S, Shamma SA, Kanold PO 2010 Dichotomy of functional organization in the mouse auditory cortex. Nat Neurosci. 13:361–368. 20118924 10.1038/nn.2490 PMC2866453

[bhx295C4] Barnstedt O, Keating P, Weissenberger Y, King AJ, Dahmen JC 2015 Functional microarchitecture of the mouse dorsal inferior colliculus revealed through *in vivo* two-photon calcium imaging. J Neurosci. 31:10927–10939. 10.1523/JNEUROSCI.0103-15.2015 PMC4524970 26245957

[bhx295C5] Belliveau LA, Lyamzin DR, Lesica NA 2014 The neural representation of interaural differences in gerbils is transformed from midbrain to cortex. J Neurosci. 34:16796–16808. 25505332 10.1523/JNEUROSCI.2432-14.2014 PMC4261102

[bhx295C6] Campbell RAA, King AJ, Nodal FR, Schnupp JWH, Carlile S, Doubell TP 2008 Virtual adult ears reveal the roles of acoustical factors and experience in auditory space map development. J Neurosci. 28:11 557–11 570. 10.1523/JNEUROSCI.0545-08.2008 PMC2656355 18987192

[bhx295C7] Campbell RAA, Schnupp JWH, Shial A, King AJ 2006 Binaural-level functions in ferret auditory cortex: Evidence for a continuous distribution of response properties. J Neurophysiol. 95:3742–3755. 16510777 10.1152/jn.01155.2005 PMC7116555

[bhx295C8] Chen QC, Cain D, Jen PHS 1995 Sound pressure transformation at the pinna of *Mus domesticus*. J Exp Biol. 198:2007–2023. 7595162 10.1242/jeb.198.9.2007

[bhx295C9] Chen TW, Wardill TJ, Sun Y, Pulver SR, Renninger SL, Baohan A, Schreiter ER, Kerr RA, Orger MB, Jayaraman V, et al 2013 Ultrasensitive fluorescent proteins for imaging neuronal activity. Nature. 499:295–300. 23868258 10.1038/nature12354 PMC3777791

[bhx295C10] Chen X, Leischner U, Rochefort NL, Nelken I, Konnerth A 2011 Functional mapping of single spines in cortical neurons in vivo. Nature. 475:501–505. 21706031 10.1038/nature10193

[bhx295C11] Dyer EL, Studer C, Robinson JT, Baraniuk RG 2013 A robust and efficient method to recover neuronal events from noisy and corrupted data. IEEE/EMBS Conf Neural Eng NER. 593–596.

[bhx295C12] Duda RO, Martens WL 1998 Range dependence of the response of a spherical head model. JASA. 104:3048–3058.

[bhx295C13] Ehret G, Dreyer A 1984 Localization of tones and noise in the horizontal plane by unrestrained house mice (*Mus musculus*). J Exp Biol. 109:163–174. 6736861 10.1242/jeb.109.1.163

[bhx295C14] Goldberg JM, Brown PB 1969 Response of binaural neurons of dog superior olivary complex to dichotic tonal stimuli: some physiological mechanisms of sound localization. J Neurophysiol. 32:613–636. 5810617 10.1152/jn.1969.32.4.613

[bhx295C15] Goodman DFM, Benichoux V, Brette R 2013 Decoding neural responses to temporal cues for sound localization. eLife. 2:e01312. 24302571 10.7554/eLife.01312 PMC3844708

[bhx295C16] Guizar-Sicairos M, Thurman ST, Fienup JR 2008 Efficient subpixel image registration algorithms. Opt Lett. 33:156–158. 18197224 10.1364/ol.33.000156

[bhx295C17] Guo W, Chambers AR, Darrow KN, Hancock KE, Shinn-Cunningham BG, Polley DB 2012 Robustness of cortical topography across fields, laminae, anesthetic states, and neurophysiological signal types. J Neurosci. 32:9159–9172. 22764225 10.1523/JNEUROSCI.0065-12.2012 PMC3402176

[bhx295C18] Hackett TA, Barkat TR, O’Brien BM, Hensch TK, Polley DB 2011 Linking topography to tonotopy in the mouse auditory thalamocortical circuit. J Neurosci. 31:2983–2995. 21414920 10.1523/JNEUROSCI.5333-10.2011 PMC3073837

[bhx295C19] Heffner RS, Heffner HE 2007 Hearing ranges of laboratory animals. J Am Assoc Lab Anim Sci. 46:11–13. 17203911

[bhx295C20] Heffner RS, Heffner HE, Kearns D, Vogel J, Koay G 1994 Sound localization in chinchillas. I: Left/right discriminations. Hear Res. 80:247–257. 7896583 10.1016/0378-5955(94)90116-3

[bhx295C21] Heffner RS, Koay G, Heffner HE 2001 Sound localization acuity changes with age in C57BL/6J mice In: Willott JF, editor Handbook of mouse auditory research: from behavior to moleculat biology. New York: CRC Press p. 31–35.

[bhx295C22] Higgins NC, McLaughlin SA, Rinne T, Stecker GC 2017 Evidence for cue-independent spatial representation in the human auditory cortex during active listening. Proc Nat Acad Sci USA. 144:E7602–E7611. 10.1073/pnas.1707522114 PMC5594681 28827357

[bhx295C23] Higgins NC, Storace DA, Escabi MA, Read HL 2010 Specialization of binaural responses in ventral auditory cortices. J Neurosci. 30:14522–14532. 20980610 10.1523/JNEUROSCI.2561-10.2010 PMC3842487

[bhx295C24] Imig TJ, Adrian HO 1977 Binaural columns in the primary field of cat auditory cortex. Brain Res. 138:241–257. 589474 10.1016/0006-8993(77)90743-0

[bhx295C25] Imig TJ, Irons WA, Samson FR 1990 Single-unit selectivity to azimuthal direction and sound pressure level of noise bursts in cat high-frequency primary auditory cortex. J Neurophysiol. 63:1448–1466. 2358885 10.1152/jn.1990.63.6.1448

[bhx295C26] Irvine DRF, Rajan R, Aitkin LM 1996 Sensitivity to interaural intensity differences of neurons in primary auditory cortex of the cat. I. Types of sensitivity and effects of variations in sound pressure level. J Neurophysiol. 75:75–96. 8822543 10.1152/jn.1996.75.1.75

[bhx295C27] Ison JR, Allen PD, O’Neill WE 2007 Age-related hearing loss in C57BL/6J mice has both frequency-specific and non-frequency-specific components that produce a hyperacusis-like exaggeration of the acoustic startle reflex. J Assoc Res Otolaryngol. 8:539–550. 17952509 10.1007/s10162-007-0098-3 PMC2538342

[bhx295C28] Issa JB, Haeffele BD, Agarwal A, Bergles DE, Young ED, Yue DT 2014 Multiscale optical Ca^2+^ imaging of tonal organization in mouse auditory cortex. Neuron. 83:944–959. 25088366 10.1016/j.neuron.2014.07.009 PMC4242551

[bhx295C29] Joachimsthaler B, Uhlmann M, Miller F, Ehret G, Kurt S 2014 Quantitative analysis of neuronal response properties in primary and higher-order auditory cortical fields of awake house mice (*Mus musculus*). Eur J Neurosci. 39:904–918. 24506843 10.1111/ejn.12478 PMC4264920

[bhx295C30] Kanold PO, Nelken I, Polly DB 2014 Local versus global scales of organization in auditory cortex. Trends Neurosci. 37:502–510. 25002236 10.1016/j.tins.2014.06.003 PMC4152386

[bhx295C31] Kara P, Boyd JD 2009 A micro-architecture for binocular disparity and ocular dominance in visual cortex. Nature. 458:627–631. 19158677 10.1038/nature07721 PMC2700034

[bhx295C32] Keating P, Dahmen JC, King AJ 2015 Complementary adaptive processes contribute to the developmental plasticity of spatial hearing. Nat Neurosci. 18:185–187. 25581359 10.1038/nn.3914 PMC4338598

[bhx295C33] Kelly JB, Judge PW 1994 Binaural organization of primary auditory cortex in the ferret (*Mustela putorius*). J Neurophysiol. 71:904–913. 8201431 10.1152/jn.1994.71.3.904

[bhx295C34] Kelly JB, Sally SL 1988 Organization of auditory cortex in the albino rat: binaural response properties. J Neurophysiol. 59:1756–1769. 3404203 10.1152/jn.1988.59.6.1756

[bhx295C35] Kerlin AM, Andermann ML, Berezovskii VK, Reid RC 2010 Broadly tuned response properties of diverse inhibitory neuron subtypes in mouse visual cortex. Neuron. 67:858–871. 20826316 10.1016/j.neuron.2010.08.002 PMC3327881

[bhx295C36] Kitzes L 2008 Binaural interactions shape binaural response structures and frequency response functions in primary auditory cortex. Hear Res. 238:68–76. 18295994 10.1016/j.heares.2008.01.003

[bhx295C37] Kugler S, Kilic E, Bahr M 2003 Human synapsin 1 gene promoter confers highly neuron-specific long-term transgene expression from an adenoviral vector in the adult rat brain depending on the transduced area. Gene Ther. 10:337–347. 12595892 10.1038/sj.gt.3301905

[bhx295C39] Kyweriga M, Stewart W, Wehr M 2014 Neuronal interaural level difference response shifts are level-dependent in the rat auditory cortex. J Neurophysiol. 111:930–938. 24335208 10.1152/jn.00648.2013 PMC3949225

[bhx295C40] Lauer AM, Slee SJ, May BJ 2011 Acoustic basis of directional acuity in laboratory mice. J Assoc Res Otolaryngol. 12:633–645. 21717290 10.1007/s10162-011-0279-y PMC3173556

[bhx295C41] Linden JF, Liu RC, Sahani M, Schreiner CE, Merzenich MM 2003 Spectrotemporal structure of receptive fields in areas AI and AAF of mouse auditory cortex. J Neurophysiol. 90:2660–2675. 12815016 10.1152/jn.00751.2002

[bhx295C42] Mianné J, Chessum L, Kumar S, Aguilar C, Codner G, Hutchison M, Parker A, Mallon AM, Wells S, Simon MM, et al 2016 Correction of the auditory phenotype in C57BL/6N mice via CRISPR/Cas9-mediated homology directed repair. Genome Med. 8:16. 26876963 10.1186/s13073-016-0273-4 PMC4753642

[bhx295C43] Mickey BJ, Middlebrooks JC 2003 Representation of auditory space by cortical neurons in awake cats. J Neurosci. 23:8649–8663. 14507964 10.1523/JNEUROSCI.23-25-08649.2003 PMC6740412

[bhx295C44] Middlebrooks JC, Dykes RW, Merzenich MM 1980 Binaural response-specific bands in primary auditory cortex of the cat: topographical organization orthogonal to isofrequency contours. Brain Res. 181:31–48. 7350963 10.1016/0006-8993(80)91257-3

[bhx295C45] Middlebrooks JC, Pettigrew J 1981 Functional classes of neurons in primary auditory cortex of the cat distinguished by sensitivity to sound location. J Neurosci. 1:107–120. 7346555 10.1523/JNEUROSCI.01-01-00107.1981 PMC6564160

[bhx295C46] Middlebrooks JC, Xu L, Eddins AC, Green DM 1998 Codes for sound-source location in nontonotopic auditory cortex. J Neurophysiol. 80:863–881. 9705474 10.1152/jn.1998.80.2.863

[bhx295C47] Middlebrooks JC, Xu L, Furukawa S, Macpherson EA 2002 Cortical neurons that localize sounds. Neuroscientist. 1:73–83. 10.1177/107385840200800112 11843102

[bhx295C48] Mills AW 1958 On the minimum audible angle. J Acous Soc Am. 30:237–246.

[bhx295C49] Mlynarski W, Jost J 2014 Statistics of natural binaural sounds. PLoS One. 9:e108968. 25285658 10.1371/journal.pone.0108968 PMC4186785

[bhx295C50] Mrsic-Flogel TDT, King AJ, Schnupp JWH 2005 Encoding of virtual acoustic space stimuli by neurons in ferret primary auditory cortex. J Neurophysiol. 93:3489–3503. 15659534 10.1152/jn.00748.2004

[bhx295C51] Nakamoto KT, Zhang J, Kitze LM 2004 Response patterns along an isofrequency contour in cat primary auditory cortex (AI) to stimuli varying in average and interaural levels. J Neurophysiol. 91:118–135. 14523080 10.1152/jn.00171.2003

[bhx295C52] Nelken I, Chechik G, Mrsic-Flogel TD, King AJ, Schnupp JW 2005 Encoding stimulus information by spike numbers and mean response time in primary auditory cortex. J Comp Neurosci. 19:199–221. 10.1007/s10827-005-1739-3 16133819

[bhx295C53] Ohki K, Reid RC 2007 Specificity and randomness in the visual cortex. Curr Opin Neurobiol. 17:401–407. 17720489 10.1016/j.conb.2007.07.007 PMC2951601

[bhx295C54] Parsons CH, Lanyon RG, Schnupp JW, King AJ 1999 Effects of altering spectral cues in infancy on horizontal and vertical sound localization by adult ferrets. J Neurophysiol. 82:2294–2309. 10561407 10.1152/jn.1999.82.5.2294

[bhx295C55] Phillips DP, Irvine DR 1983 Some features of binaural input to single neurons in physiologically defined area AI of cat cerebral cortex. J Neurophysiol. 49:383–395. 6834083 10.1152/jn.1983.49.2.383

[bhx295C56] Phillips DP, Judge PW, Kelly JB 1988 Primary auditory cortex in the ferret (*Mustela putorius*): neural response properties and topographic organization. Brain Res. 443:281–294. 3359271 10.1016/0006-8993(88)91622-8

[bhx295C57] Polley DB, Thompson JH, Guo W 2013 Brief hearing loss disrupts binaural integration during two early critical periods of auditory cortex development. Nat Comm. 4:2547. 10.1038/ncomms3547 PMC4131765 24077484

[bhx295C58] Rajan R, Aitkin LM, Irvine DRF, McKay J 1990 Azimuthal sensitivity of neurons in primary auditory cortex of cars. I. Types of sensitivity and the effects of variations in stimulus parameters. J Neurophysiol. 64:872–887. 2230931 10.1152/jn.1990.64.3.872

[bhx295C59] Razak KA 2011 Systematic representation of sound locations in the primary auditory cortex. J Neurosci. 31:13848–13859. 21957247 10.1523/JNEUROSCI.1937-11.2011 PMC3219787

[bhx295C60] Reale R, Kettner R 1986 Topography of binaural organization in primary auditory cortex of the cat: effects of changing interaural intensity. J Neurophysiol. 56:663–682. 3783214 10.1152/jn.1986.56.3.663

[bhx295C61] Recanzone GH, Beckerman NS 2004 Effects of intensity and location on sound location discrimination in macaque monkeys. Hear Res. 198:116–124. 15567608 10.1016/j.heares.2004.07.017

[bhx295C62] Recanzone GH, Schreiner CE, Sutter ML, Beitel RE, Merzenich MM 1999 Functional organization of spectral receptive fields in the primary auditory cortex of the owl monkey. J Comp Neurol. 415:460–481. 10570456 10.1002/(sici)1096-9861(19991227)415:4<460::aid-cne4>3.0.co;2-f

[bhx295C63] Reser DH, Fishman YI, Arezzo JC, Steinschneider M 2000 Binaural interactions in primary auditory cortex of the awake macaque. Cereb Cortex. 10:574–584. 10859135 10.1093/cercor/10.6.574

[bhx295C64] Rothschild G, Nelken I, Mizrahi A 2010 Functional organization and population dynamics in the mouse primary auditory cortex. Nat Neurosci. 13:353–360. 20118927 10.1038/nn.2484

[bhx295C65] Rutkowski RG, Wallace MN, Shackleton TM, Palmer AR 2000 Organisation of binaural interactions in the primary and dorsocaudal fields of the guinea pig auditory cortex. Hear Res. 145:177–189. 10867291 10.1016/s0378-5955(00)00087-3

[bhx295C66] Schnupp JW, garcia-Lazaro JA, Lesica NA 2015 Periodotopy in the gerbil inferior colliculus: local clustering rather than a gradient map. Front Neural Circuits. 9:37. 26379508 10.3389/fncir.2015.00037 PMC4550179

[bhx295C67] Schreiner CE, Cynader MS 1984 Basic functional organization of secondary auditory cortical field (AII) of the cat. J Neurophysiol. 51:1284–1305. 6737031 10.1152/jn.1984.51.6.1284

[bhx295C68] Schreiner CE, Winer JA 2007 Auditory cortex mapmaking: principles, projections, and plasticity. Neuron. 56:356–365. 17964251 10.1016/j.neuron.2007.10.013 PMC2412907

[bhx295C69] Shoham S, O’Conner D, Segev R 2006 How silent is the brain: is there a “dark matter” problem in neuroscience? J Comp Physiol A Neuroethol Sens Neural Behav Physiol. 192:777–784. 16550391 10.1007/s00359-006-0117-6

[bhx295C70] Stecker GC, Harrington IA, Middlebrooks JC 2005 Location coding by opponent neural populations in the auditory cortex. PloS Biol. 3:e78. 15736980 10.1371/journal.pbio.0030078 PMC1044834

[bhx295C71] Stecker GC, McLaughlin SA, Higgins NC 2015 Monaural and binaural contributions to interaural-level-difference sensitivity in human auditory cortex. Neuroimage. 120:456–466. 26163805 10.1016/j.neuroimage.2015.07.007 PMC4589528

[bhx295C72] Stecker GC, Middlebrooks JC 2003 Distributed coding of sound locations in the auditory cortex. Biol Cybern. 89:341–349. 14669014 10.1007/s00422-003-0439-1

[bhx295C73] Stiebler I, Neulist R, Fichtel I, Ehret G 1997 The auditory cortex of the house mouse: left-right differences, tonotopic organization and quantitative analysis of frequency representation. J Comp Physiol A Neuroethol Sens Neural Behav Physiol. 181:559–571. 10.1007/s003590050140 9449817

[bhx295C74] Tian L, Hires SA, Mao T, Hiber D, Chiappe ME, Chalasani SH, Petreanu L, Akerboom J, McKinney SA, Schreiter ER, et al 2009 Imaging neural activity in worms, flies and mice with improved GCaMP calcium indicators. Nat Methods. 6:875–881. 19898485 10.1038/nmeth.1398 PMC2858873

[bhx295C75] Tollin DJ, Yin TC 2005 Interaural phase and level difference sensitivity in low-frequency neurons in lateral superior olive. J Neurosci. 25:10648–10657. 16291937 10.1523/JNEUROSCI.1609-05.2005 PMC1449742

[bhx295C76] Tsukano H, Horie M, Bo T, Uchimura A, Hishida R, Kudoh M, Takahashi K, Takebayashi H, Shibuki K 2015 Delineation of a frequency-organized region isolated from the mouse primary auditory cortex. J Neurophysiol. 113:2900–2920. 25695649 10.1152/jn.00932.2014 PMC4416634

[bhx295C77] Tsukano H, Horie M, Hishida R, Takahashi K, Takebayashi H, Shibuki K 2016 Quantitative map of multiple auditory cortical regions with a stereotaxic fine-scale atlas of the mouse brain. Sci Rep. 6:22315. 26924462 10.1038/srep22315 PMC4770424

[bhx295C78] Vasquez-Lopez SA, Weissenberger Y, Lohse M, Keating P, King AJ, Dahmen JC 2017 Thalamic input to auditory cortex is locally heterogeneous but globally tonotopic. eLife. 6:e25141. 28891466 10.7554/eLife.25141 PMC5614559

[bhx295C79] Wesolek CM, Koay G, Heffner RS, Heffner HE 2010 Laboratory rats (*Rattus norvegicus*) do not use binaural phase differences to localize sound. Hear Res. 265:54–62. 20184949 10.1016/j.heares.2010.02.011

[bhx295C80] White EL 1989 Cortical circuits: synaptic organization of the cerebral cortex–structure, function and theory. Boston (MA): Birkhauser.

[bhx295C81] Willott JF, Aitkin LM, McFadden SL 1993 Plasticity of auditory cortex associated with sensorineural hearing loss in adult C57BL/6J mice. J Comp Neurol. 329:402–411. 8459051 10.1002/cne.903290310

[bhx295C82] Winkowski DE, Kanold PO 2013 Laminar transformation of frequency organization in auditory cortex. J Neurosci. 33:1498–1508. 23345224 10.1523/JNEUROSCI.3101-12.2013 PMC3783029

[bhx295C83] Xiong XR, Liang F, Li H, Mesik L, Zhang KK, Polley DB, Tao HW, Xiao Z, Zhang LI 2013 Interaural level difference-dependent gain control and synaptic scaling underlying binaural computation. Neuron. 79:738–753. 23972599 10.1016/j.neuron.2013.06.012 PMC3755964

[bhx295C84] Yao JD, Bremen P, Middlebrooks JC 2013 Rat primary auditory cortex is tuned exclusively to the contralateral hemifield. J Neurophysiol. 110:2140–2151. 23945782 10.1152/jn.00219.2013 PMC3841933

[bhx295C85] Zhang J, Nakamoto KT, Kitzes LM 2004 Binaural interaction revisited in the cat primary auditory cortex. J Neurophysiol. 91:101–117. 14507982 10.1152/jn.00166.2003

[bhx295C86] Zhou Y, Wang X 2012 Level dependence of spatial processing in the primate auditory cortex. J Neurophysiol. 108:810–826. 22592309 10.1152/jn.00500.2011 PMC3424089

