# Supplementary material for: Local and Global Spatial Organization of Interaural Level Difference and Frequency Preferences in Auditory Cortex
Source: Cereb Cortex. 2017 Nov 9;28(1):350–69. doi: 10.1093/cercor/bhx295 (PMC5991210; doi:10.1093/cercor/bhx295)
Supplement: Supplementary Data [file bhx295suppl_1.zip › Supplementary_Legends.docx]

**Supplementary Figure 1.** (*A)* Individual (light blue) and mean (dark blue) calcium responses of 10 neurons in the same imaging field to noise bursts presented at 60 dB ABL. Each row represents a different neuron. The position and width of the gray bars indicate stimulus onset and duration. Plots to the right represent the ILD response areas of the same 10 neurons (and therefore exclude the responses to monaural stimulation). The black boxes highlight for neuron 3 the responses to noise bursts at different ILDs presented at 60 dB ABL. (*B*) ILD response area plots from 20 further neurons imaged in the same mouse.

**Supplementary Figure 2.** Calcium traces (top row) and inferred spike trains (bottom row) from 4 example neurons. (*A*) Two neurons in which GCaMP6m was expressed via AAV injection. (*C*) Two neurons in which GCaMP6f was constitutively expressed. Insets to the right of these plots show 10-second segments of the traces, with their timing indicated by the dashed boxes in the plots on the left. Similar ILD response functions from 3 representative neurons expressing GCaMP6m (*B*) and 3 expressing GCaMP6f (*D*) were obtained from calcium signals (black lines) and inferred spikes (gray lines).

**Supplementary Figure 3.** (*A)* Mean percentages of neurons preferring different ILD values at 40 dB ABL (left) and at 80 dB ABL (right) across all mice injected with GCaMP6m. (*B)* Mean position of the steepest ILD response function slopes at 40 dB ABL (left) and at 80 dB ABL (right).

**Supplementary Figure 4.** Percentages of responsive neurons assigned to each binaural category, averaged across mice injected with GCaMP6m (mean ± sem), at 40 dB ABL (above) and 80 dB ABL (below).

**Supplementary Figure 5.** *(A)* Three-dimensional plots of the relative cortical location of neurons in the same mice shown in Figs. 6*B, 7B* and 10. The medial border of the ILD response function was calculated as the ILD eliciting 50% of the maximal response for a given neuron. This was calculated for neurons demonstrating either a contralateral ear preference (colored circles on the plot), ipsilateral ear preference (diamonds), or a single peaked response (squares) (based on Razak 2011). The medial ILD border was calculated on the ipsilateral side of single peak ILD functions. The resulting medial ILD border for each neuron is color coded on the plot. Noise-responsive neurons that did not show significant ILD sensitivity (white circles) and neurons with multi-peaked ILD response functions (crosses) are also shown. *(B)* Medial ILD borders of neurons are shown as in *(A)*, for each of the 3 transgenic mice.
