# Supplementary figures and images for: Local and Global Spatial Organization of Interaural Level Difference and Frequency Preferences in Auditory Cortex
Source: Cereb Cortex. 2017 Nov 9;28(1):350–69. doi: 10.1093/cercor/bhx295 (PMC5991210; doi:10.1093/cercor/bhx295)

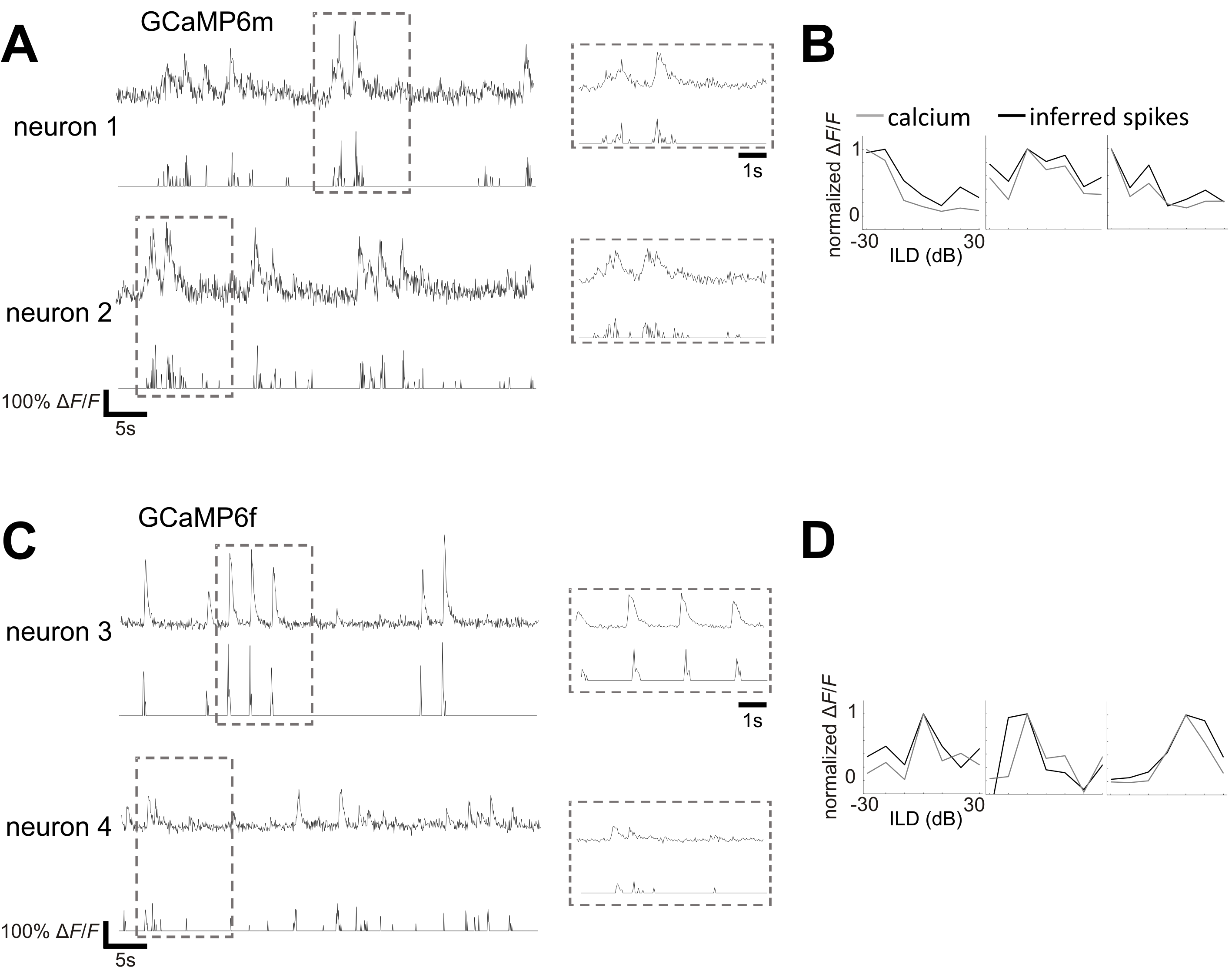

Supplement: Supplementary Data [file bhx295suppl_1.zip › SupplFig2.tif]
